# Unveiling the Multifaceted Capabilities of Endophytic *Aspergillus flavus* Isolated from *Annona squamosa* Fruit Peels against *Staphylococcus* Isolates and HCoV 229E—In Vitro and In Silico Investigations

**DOI:** 10.3390/ph17050656

**Published:** 2024-05-19

**Authors:** Noha Fathallah, Wafaa M. Elkady, Sara A. Zahran, Khaled M. Darwish, Sameh S. Elhady, Yasmin A. Elkhawas

**Affiliations:** 1 Department of Pharmacognosy and Medicinal Plants, Faculty of Pharmacy, Future University in Egypt, Cairo 11835, Egypt; welkady@fue.edu.eg; 2 Department of Microbiology and Immunology, Faculty of Pharmacy, Future University in Egypt, Cairo 11835, Egypt; sara.zahran@fue.edu.eg; 3 Department of Medicinal Chemistry, Faculty of Pharmacy, Suez Canal University, Ismailia 41522, Egypt; khaled_darwish@pharm.suez.edu.eg; 4 King Abdulaziz University Herbarium, Faculty of Science, King Abdulaziz University, Jeddah 21589, Saudi Arabia; ssahmed@kau.edu.sa; 5 Department of Biological Sciences, Faculty of Science, King Abdulaziz University, Jeddah 21589, Saudi Arabia; 6 Center for Artificial Intelligence in Precision Medicines, King Abdulaziz University, Jeddah 21589, Saudi Arabia

**Keywords:** *Annona squamosa* L., endophytic fungi, MRSA, antiviral, ADME prediction, public health, drug discovery, molecular docking-coupled dynamics simulation

## Abstract

Recently, there has been a surge towards searching for primitive treatment strategies to discover novel therapeutic approaches against multi-drug-resistant pathogens. Endophytes are considered unexplored yet perpetual sources of several secondary metabolites with therapeutic significance. This study aims to isolate and identify the endophytic fungi from *Annona squamosa* L. fruit peels using morphological, microscopical, and transcribed spacer (ITS-rDNA) sequence analysis; extract the fungus’s secondary metabolites by ethyl acetate; investigate the chemical profile using UPLC/MS; and evaluate the potential antibacterial, antibiofilm, and antiviral activities. An endophytic fungus was isolated and identified as *Aspergillus flavus* L. from the fruit peels. The UPLC/MS revealed seven compounds with various chemical classes. The antimicrobial activity of the fungal ethyl acetate extract (FEA) was investigated against different Gram-positive and Gram-negative standard strains, in addition to resistant clinical isolates using the agar diffusion method. The CPE-inhibition assay was used to identify the potential antiviral activity of the crude fungal extract against low pathogenic human coronavirus (HCoV 229E). Selective Gram-positive antibacterial and antibiofilm activities were evident, demonstrating pronounced efficacy against both methicillin-resistant *Staphylococcus aureus* (MRSA) and methicillin-sensitive *Staphylococcus aureus* (MSSA). However, the extract exhibited very weak activity against Gram-negative bacterial strains. The ethyl acetate extract of *Aspergillus flavus* L exhibited an interesting antiviral activity with a half maximal inhibitory concentration (IC_50_) value of 27.2 µg/mL against HCoV 229E. Furthermore, in silico virtual molecular docking-coupled dynamics simulation highlighted the promising affinity of the identified metabolite, orienting towards three MRSA biotargets and HCoV 229E main protease as compared to reported reference inhibitors/substrates. Finally, ADME analysis was conducted to evaluate the potential oral bioavailability of the identified metabolites.

## 1. Introduction

Drug-resistant bacteria and fungi are thought to pose a global health risk. Microorganisms that produce biofilms present one of the challenges that scientists face today due to their unique capacity to modify their immediate environs through intriguing phenotypic plasticity that involves changes in their physiology and their resistance to antimicrobial agents [1]. Since the late 1970s, (MRSA) infections have been linked to multiple hospital outbreaks and are a major cause of morbidity and mortality among hospitalized patients worldwide. In comparison to other African nations as well as countries in the southern and eastern Mediterranean, Egypt had the highest MRSA rates among clinical isolates of *S. aureus* [2]. Another major problem facing the healthcare system in Egypt is acute respiratory infections (ARIs) which are a chief cause of morbidity and mortality among children under five, which also causes absenteeism due to respiratory symptoms among primary and preparatory school students. At the end of 2022, numerous governmental surveillances detected a surge of respiratory viruses including coronavirus [3].

Coronavirus species are known to cause human infection, one of which, HCoV 229 E, typically causes cold symptoms in immunocompetent individuals [4]; it causes mild to severe enteric, respiratory, and systemic disease in animals, poultry, and rodents, and causes common cold or pneumonia in humans. Thus, it was deemed necessary to search for potential new and promising antimicrobial and antiviral non-conventional drugs. Since the dawn of human civilization, plants have been a significant source of medicinal compounds [5,6]. Current demand for new and potent medications and other plant-based items is rising.

Drug-resistant bacteria are thought to pose a global health problem. Biofilm-forming bacteria are among the issues facing scientists today, with their special ability to alter their immediate environs by unusual phenotypical plasticity that encompasses changes in their physiology and resistance to antimicrobial treatments; Singab et al. reported that endophytes have been identified as a hidden treasure for secondary metabolites. According to previously reported data, various compounds isolated from *Aspergillus flavus* showed antimicrobial, anti-biofilm activity [7]. Khattak et al. reported that the *Aspergillus flavus* isolated compound demonstrated significant antibacterial activity against *S. aureus* [8].

It has been established after more than a century of research that the majority of plants in ecosystems —if not all of them—are symbiotic with fungal endophytes, including grass, trees, algae, and herbaceous plants [9,10]. The expression of host plant diseases can be significantly altered by non-pathogenic fungi found within plants, also known as endophytes (“endo” = within, “phyte” = plant), according to recent studies [11,12]. These fungi are valuable sources of bioactive secondary metabolites that can produce broad-spectrum antimicrobial substances [6,13].

*Annona squamosa* Linn tree, commonly known as the sugar apple, is endogenous to Egypt [14]. It yields edible fruits and is used to make both industrial and therapeutic items. *A. squamosa* Linn is currently employed as an anti-inflammatory [15], cytotoxic [16], antitumor, hepatoprotective [17], antidiabetic [18], and anti-lice agent [19]. It is associated with the presence of alkaloids, carbohydrates, tannins, fixed oils, and phenolics [20,21,22]. It was previously evaluated for its antimicrobial activity [5,23,24] and was established as a plant with a potential wide spectrum of antimicrobial activity. Due to the genetic transmission or co-evolution between the endophyte and host, some of the fruits’ therapeutic benefits may be attributed to the endophytes [9].

On one hand, the current study aimed to isolate the endophytic fungi associated with *A. squamosa* fruits, and to identify the metabolites that may be useful by employing the UPLC/MS analytical technique, which is a rapid and affordable method of identification. On the other hand, in vitro experiments to evaluate the ethyl acetate’s antibacterial and antiviral potentials followed by a rapid prediction using preliminary computational in silico and ex silico studies are undertaken to assess the drug-like properties of those lead compounds. 

## 2. Results and Discussion

### 2.1. Isolation and Identification of Endophytic Fungi

**Figure 1:**
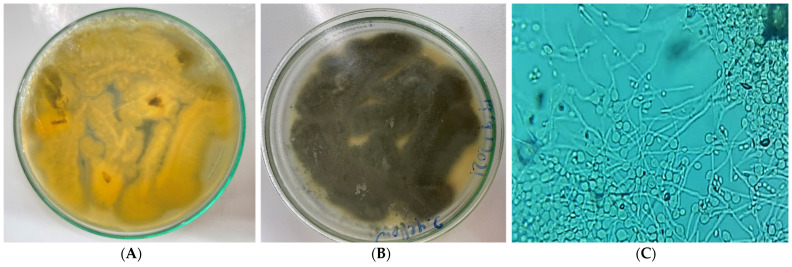
Photos of *Aspergillus flavus* fungus; (**A**,**B**) colony morphology on potato dextrose agar after 2 days of incubation; (**A**) front view, (**B**) back view. (**C**) Under microscope (1000×).

**Figure 2:**
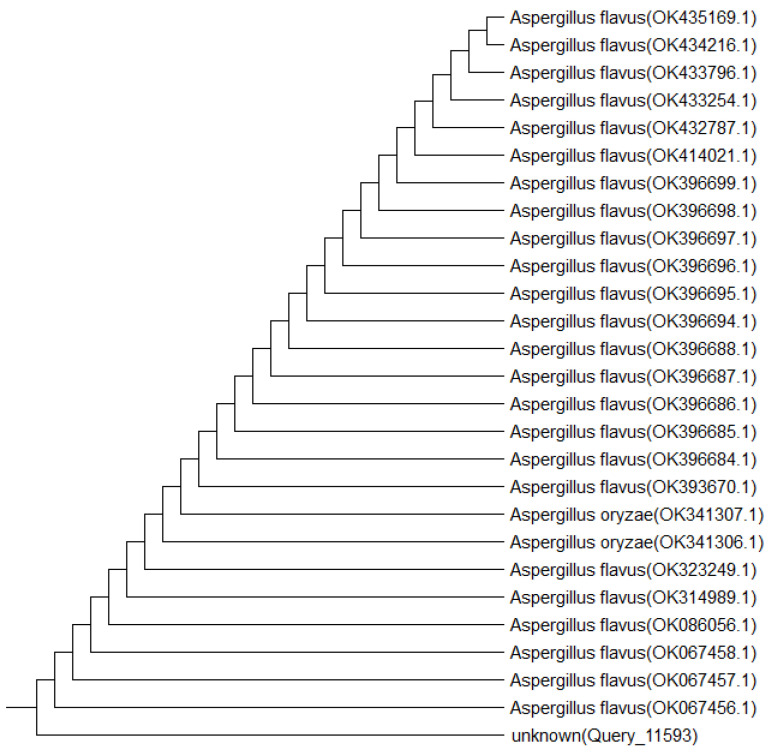
Phylogenetic tree of the isolated endophyte *Aspergillus flavus*.

In the present study, the particular fungus under investigation was the only one being successfully sub-cultured and purified through repeated culturing of the crushed *A. squamosa* L. fruits. Notably, the mother culture revealed various endophytes, yet they failed to grow upon sub-culturing. Using the morphological and microscopical features listed in Table 1 and Figure 1, the isolated purified endophyte would belong to the *Aspergillus* species. The identification was confirmed using amplification and sequencing of the internal transcribed spacer ribosomal RNA (ITS rRNA) gene. Sequence analysis showed a 99% identity with *Aspergillus flavus* (*A. flavus*) as seen in Figure 2. Upon submission, a GenBank accession number OM095472 was assigned to the ITS rRNA gene sequence.

Patil et al., Liu et al., and Ola et al. [25,26,27] previously covered the significance of the endophytic *A. flavus* isolated from several plant species. They demonstrated how it might be valuable as an antibacterial and anticancer agent. This provided us with a clue to design a study that would investigate the contribution of endophytic fungus to the previously reported activity of *A. squamosa*, as well as identify the chemicals responsible for antibacterial, antibiofilm, and antiviral activity.

Aflatoxins are common toxic active metabolites usually produced by *A. flavus*. They are known to appear in the media as yellow pigments, which could be easily visualized on the reverse side of a coconut-agar medium colony [28]; their products turn pink/plum red when exposed to ammonia vapor and usually give blue fluorescence on CAM when exposed to UV light (365 nm). Interestingly, our isolate did not produce any yellow pigments, any pink color, or any blue fluorescence upon applying the three tests; thus, it was concluded that it is a non-aflatoxigenic isolate [29].

### 2.2. Metabolic Profiling of the Ethyl Acetate Extract

**Figure 3:**
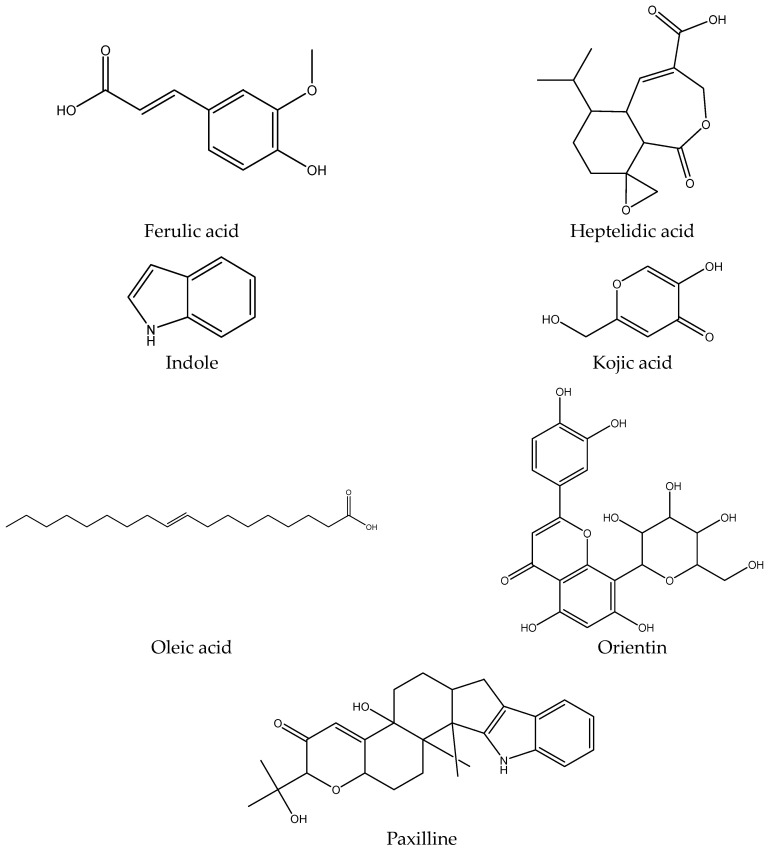
Two-dimensional structures of the identified compounds by UPLC/MS analysis (Chem-draw ultra-version 14).

To investigate the active metabolites present in the identified fungus, metabolic profiling using UPLC/MS was conducted. This approach was used as it is a sensitive and accurate method of analysis, allows for separation in a shorter development and analysis time than conventional LC/UV, and it provides a comprehensive profile of the compounds present in the extract [30]. The chromatogram represented in Figure S1 revealed seven compounds as illustrated in Table 2 and Figure 3. Most of the identified metabolites have been previously reported from various endophyte species. It was noted that the extract revealed several classes of active metabolites such as sesquiterpenoids, phenolics, fatty acids, flavonoids, and pyrones. Heptelidic acid, ferulic acid, and oleic acid were the dominant active metabolites identified with areas of 26.8%, 25.3%, and 23.2%, respectively.

On the one hand, heptelidic acid was the most dominant compound. This compound was reported before by Tanaka et al., Itoh et al., and Kim et al. as an antimalarial, antibiotic, and anticancer agent [31,32,33]. On the other hand, ferulic acid is known for its broad antimicrobial activities [34,35]. Nevertheless, the rest of the identified compounds are known for their remarkable array of biochemical and pharmacological actions [36,37], suggesting that they may significantly affect the function of various mammalian cellular systems. These results encouraged additional research on the FEA’s antiviral, antibiofilm, and antimicrobial properties.

**Table 2:** Peak assignments of the ethyl acetate extract of *A. flavus*, L via UPLC-ESI-MS/MS in negative ionization mode. (N.B. the compounds numbered according to their abundance.)

No.	Compound	Chemical Class	Molecular Formula	[M-H]^−^	Abundance	M. Weight	Ref.
1	Heptelidic acid	Sesquiterpene	C_15_H_20_O_5_	279	26.8%	280	[38,39]
2	Ferulic acid	Phenolic	C_10_H_10_O_4_	193	25.3%	194	[40,41]
3	Oleic acid	Fatty acid	C_18_H_34_O_2_	281	23.2%	282	[42]
4	Paxilline	Diterpene indole polycyclic alkaloid	C_27_H_33_NO_4_	432	8.3%	435	[43,44]
5	Indole	Alkaloid	C_8_H_7_N	116	7.4%	117	[45]
6	Orientin	Flavonoid	C_21_H_20_O_11_	446	6.4%	447	[46]
7	Kojic acid	Pyrone	C_6_H_6_O_4_	141	2%	142	[47,48]

### 2.3. Antimicrobial Potential

Endophytes’ interactions with the plants vary from antagonism to mutualism. Usually, the host plant provides the endophytes with food and protection while the latter increases the host’s resistance to herbivores, infections, as well as different abiotic stressors [49], thus it is now considered as a promising approach for discovering new potent antimicrobial agents.

The antimicrobial activity of FEA (20% *w*/*v*) was evaluated using the disc diffusion technique against a diverse panel of microbes. Notably, FEA demonstrated maximum inhibition zones against Gram-positive bacteria, specifically *S. aureus* ATCC 25923 (which is MSSA) and MRSA ATCC-700788. The inhibition zones were measured at (15 ± 0.4 mm) and (11 ± 0.7 mm), respectively, approaching the efficacy of the standard drug vancomycin, which displayed zones of inhibition at (18 ± 0.2 mm) and (13 ± 0.3 mm) against the same strains (Table 3). Interestingly, no observable inhibition zones were detected when testing FEA against Gram-negative isolates such as *Escherichia coli* ATCC 25922 and *Pseudomonas aeruginosa* ATCC 9027, as well as the *Candida albicans* ATCC 10231 strains. The results unveil a distinct selective antibacterial activity of FEA, particularly targeting Gram-positive bacteria. 

### 2.4. Minimum Inhibitory Concentration

**Figure 4:**
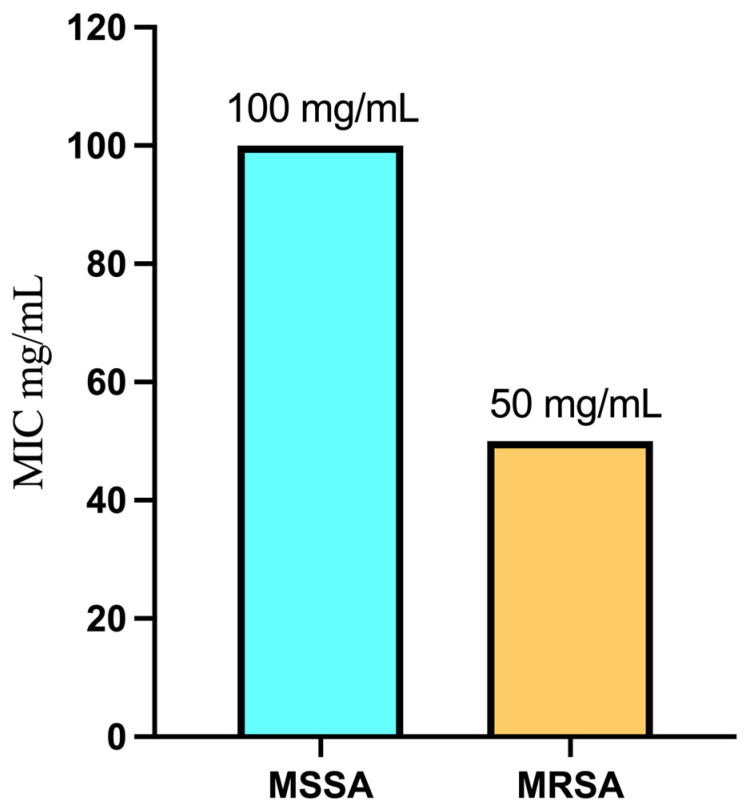
Minimum inhibitory concentration of FEA against sensitive Gram-positive strains.

Figure 4 presents a summary of the Minimum Inhibitory Concentration (MIC) values for FEA against sensitive Gram-positive strains, specifically *S. aureus* ATCC 25923 (MSSA) and MRSA ATCC-700788. The results reveal that FEA exhibited potent antimicrobial activity, with the lowest MIC recorded at 50 mg/mL for MRSA ATCC-700788. In contrast, a higher MIC value of 100 mg/mL was observed for *S. aureus* ATCC 25923 (MSSA), indicating a higher susceptibility of MRSA to the extract. Despite the higher MIC for MSSA, FEA remains effective against both *S. aureus* strains. These results highlight the potential of FEA as a natural antimicrobial agent, particularly against problematic bacterial strains such as MRSA. This could be a valuable approach to conquer *S. aureus* as this organism when compared to other microorganisms can serve as an example of the adaptive evolution of bacteria during the antibiotic era. That is because it has shown a remarkable capacity to rapidly adapt to new antibiotics by developing resistance mechanisms. Not only does the resistance mechanism involve the antibiotic’s enzymatic deactivation but also it forms biofilm which is considered a major virulence factor [50,51].

### 2.5. Antibiofilm Activity/Anti-Adhesion

#### 2.5.1. Prevention of Cell Attachment

**Figure 5:**
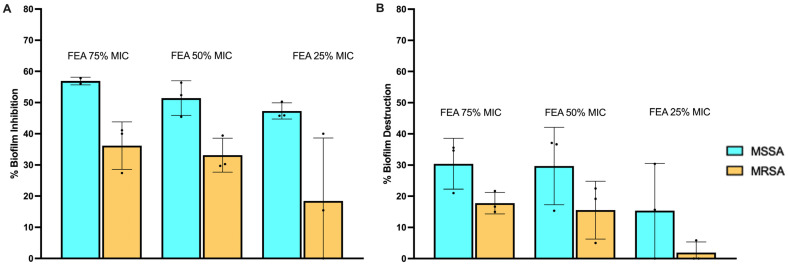
Antibiofilm activity of FEA at its sub-MIC concentrations (75, 50, and 25%) against both MSSA and MRSA isolates. (**A**) Inhibition of biofilm formation and (**B**) biofilm mass destruction. Values ranging from 0% to 50% indicate low activity, and values exceeding 50% indicate high activity. All measurements were conducted in triplicate, and the results are presented as means ± standard deviation (SD).

The effect of Sub-Minimum Inhibitory Concentration (Sub-MIC) of FEA on biofilm formation by *S. aureus* ATCC 25923 (MSSA) and MRSA ATCC-700788 is illustrated in Figure 5A. As per established criteria [52], percentage inhibition values ranging from 0 to 100% are indicative of biofilm inhibition, while values below 0% suggest the enhancement of biofilm formation. Activities surpassing the 50% inhibition threshold are considered good, while those falling between 0 and 49% are deemed poor [53]. The fungal extract displayed notable activity in preventing biofilm attachment, and the observed effects were found to be dosage-dependent. Notably, FEA exhibited effective prevention of biofilm attachment for *S. aureus* ATCC 25923 (MSSA) at concentrations of 75 and 50 mg/mL (75 and 50% MIC), surpassing the significant 50% inhibition threshold. However, for MRSA ATCC-700788, the observed suppression remained below the 50% inhibition threshold, even at the highest tested concentration of 37.5 mg/mL (75% MIC).

#### 2.5.2. Evaluating Biofilm Mass Destruction

Figure 5B illustrates the effects of the fungal extracts on destroying or reducing further development in 24 h preformed biofilms. Once again, a dose-dependent antibiofilm activity was evident. However, it is noteworthy that the ability to destroy an already-formed biofilm is not as powerful as the prevention of attachment. In this context, all observed activities exhibited poor biofilm inhibition, falling below 50%. Across all concentrations of FEA, the inhibitory effects were consistently more pronounced against *S. aureus* ATCC 25923 (MSSA) compared to MRSA ATCC-700788.

These findings suggest that while the fungal extract may effectively prevent initial biofilm attachment, particularly against MSSA, its ability to eradicate established biofilms is less potent.

### 2.6. Antiviral Activity of Crude Extract

FEA demonstrated noteworthy antiviral activity, as evidenced by a CC_50_ value of 46.38 µg/mL, and (IC_50_) value of 27.2 µg/mL against low pathogenic coronavirus (HCoV 229E), indicating that the extract effectively inhibits viral replication at a relatively low concentration. However, the calculated selectivity index (SI = CC_50_/IC_50_) of approximately 2 implies a narrow therapeutic window for the extract, raising concerns about its safety profile [54]. It was reported by Hasöksüz et al. [4] that the virulence and pathophysiology mechanisms of CoVs may be attributed to nonstructural proteins which block the host’s innate immune response and structural proteins that play a crucial role in promoting viral assembly and release. FEA established a distinct potency against the HCoV 229E virus which may indicate that its compounds interfere with the function of the nonstructural protein or affect the envelop formation by hindering the structural proteins. Overall, while the antiviral potential of the FEA is promising, further studies are needed to optimize its safety profile and evaluate its efficacy in vivo before considering it as a potential antiviral agent for clinical use. 

### 2.7. Online Software Swiss ADME Prediction (Boiled Egg Method and Lipinski’s Rule of Five)

As discussed by [55], it is commonly known that ADME data, whether computationally predicted or empirically observed, provide important information about how a drug will eventually be absorbed, distributed, metabolized, or excreted by the body. While there are other ways to administer drugs, oral dosage is strongly recommended for patient comfort and compliance. An important criterion for decision making at different stages of the discovery process is the early calculation of oral bioavailability, which is defined as the fraction of the dose that enters the bloodstream following oral administration

**Figure 6:**
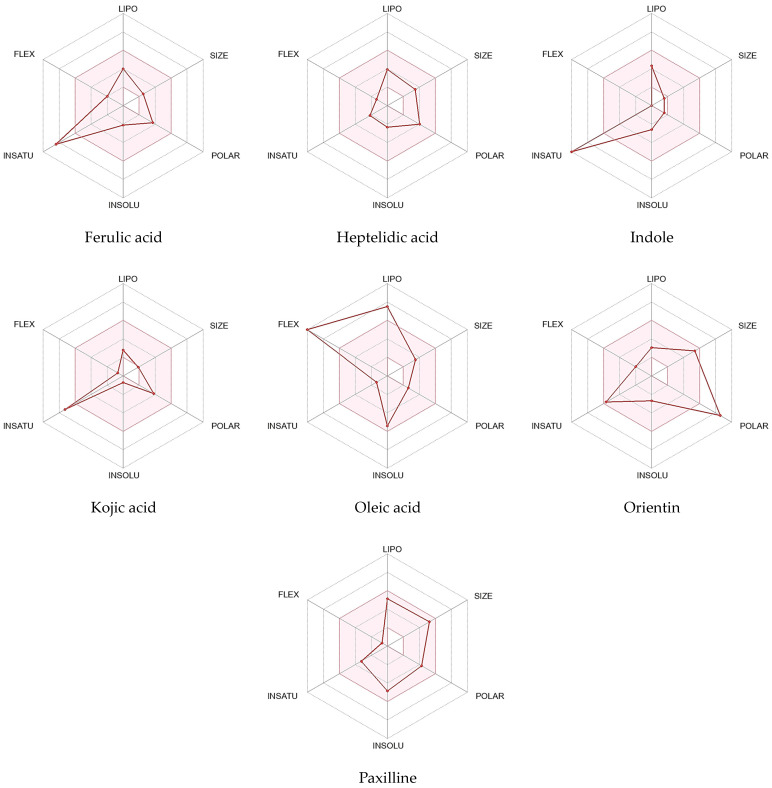
Compounds’ polarity, lipophilicity, solubility, flexibility, and saturation are represented on the radar map as POLAR, LIPO, INSOLU, and IN-SATU, respectively. The magenta area represents the optimal range for each molecular property. Solubility: log S < 6; sizes: MW between 150 and 500 g/mol; saturation: fraction of carbons in the sp3 hybridization > 0.25; polarity: TPSA between 20 and 130 Å; flexibility: <9 rotatable bonds. Lipophilicity: XLOGP3 between −0.7 and +5.0.

**Figure 7:**
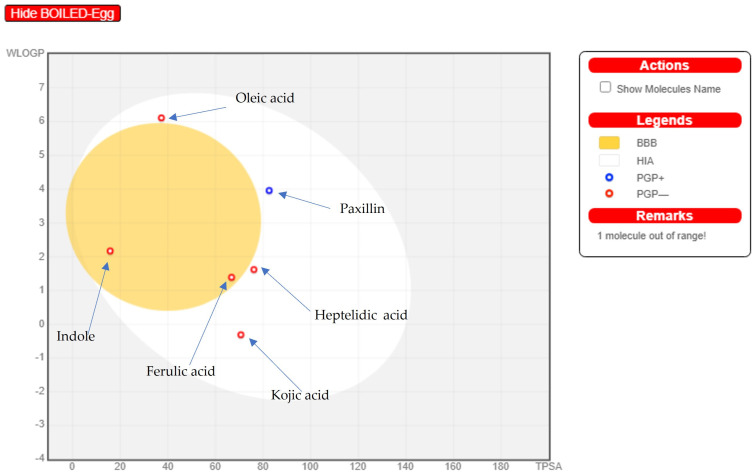
Compounds evaluation using Boiled Egg Method for BBB and GIT absorption. N.B: Boiled egg 2D graphical representation; the yolk area represents the molecules that can passively permeate through the blood–brain barrier (BBB); the molecules located in the white region are predicted to be passively absorbed by the gastrointestinal (GI) tract. Ferulic acid TPSA: 66.76 Å^2^, WLOGP 1.39; heptelidic acid TPSA: 76.13 Å^2^, WLOGP 1.62; indole TPSA: 15.79 Å^2^, WLOGP 2.17; kojic acid TPSA: 70.67 Å^2^, WLOGP −0.31; oleic acid TPSA: 37.30 Å^2^, WLOGP 6.11; paxilline 82.55 Å^2^, WLOGP 3.96.

Identified compounds’ physicochemical properties were assessed using Lipinski’s rule of five and ADME, which aid in the approval process for prospective compounds for use in biological systems [54,56]. As can be seen in Table 4, most of the compounds met Lipinski’s requirements to become an oral medication. However, Orientin exhibited two violations in the number of hydrogen bond donors (>5) and acceptors (>10). Nevertheless, as can be seen in Figure 6, the radar plot bioavailability technique predicted that two compounds, namely heptelidic acid and paxilline, can become completely orally bioavailable as all their parameters were found in the pink bioavailable area. Yet five compounds exhibited deviation in one parameter. Ferulic acid, indole, and kojic acid showed INSATU parameter deviancy while oleic acid and orientin were offshoots of the vertex in flexibility and polarity parameters, respectively. The EGG-BOILED model facilitates the intuitive assessment of the white part of passive gastrointestinal absorption (HIA) and the yellow part of brain penetration (BBB). The physicochemical zone containing chemicals expected to have significant intestinal absorption is known as the “grey region”. Regarding the compounds, as observed in Figure 7, two of them were found in the yolk area, namely ferulic acid and indole, while four were in the white zone, namely kojic acid, paxilline, oleic acid, and heptelidic acid. Orientin TPSA 201.28 Å^2^ was out of the threshold area [57]. Additionally, most of the compounds were predicted by software as non-substrates (PGP−) of the permeability glycoprotein (PGP) being shown in red circles. Contrarily, only paxilline was shown as a blue circle corresponding to a substrate (PGP +) of glycoprotein permeability.

### 2.8. In Silico Investigation: Molecular Docking Simulation

In silico studies are performed as they are considered an effective approach for determining drug protein-bound structures and binding affinities down to their molecular levels. Driven by the obtained antibacterial and antiviral activities, we were interested in investigating such activities down to the molecular levels. The identified phytochemicals were evaluated for their binding affinities and interactions towards several potential biotargets highlighting their antibacterial and antiviral activities. In terms of activity against *S. aureus* and its methicillin-resistant strain (MRSA), the molecular aspects of the identified phytochemicals’ binding affinity with several targets involved in peptidoglycan biosynthesis were investigated. Most of the marketed drugs commonly applied for managing *S. aureus* and MRSA are those designed for hampering its peptidoglycan biosynthesis, the crucial component of the bacterial cell wall [58]. Typically, peptidoglycans confer the bacterial cell wall’s flexibility and robustness and thus interfering with their biosynthesis would mediate bactericidal actions [59]. The presented study explored the potential of identified phytochemicals to block relevant steps across peptidoglycan biosynthesis. The bacterial MurE ligase is typically involved within the cytosolic biosynthesis of peptidoglycan’s starting units: UDP-*N*-acetylglucosamine for producing the UDP-*N*-acetylmuramyl-multi-peptide product [60]. The final stage involved the formation of linear peptidoglycans via the _DD_-transpeptidase catalytic activity of penicillin-binding proteins (e.g., PBP2a) following the transfer of the disaccharide pentapeptides to the cell membrane’s outer surface [61]. The development of multi-target drugs has been considered advantageous for circumventing the most common antibiotic resistance mechanism which is the target mutations [62,63].

Out of an evolutionary concept, targeting multiple independent paths for inhibitions is unlikely to allow bacteria to develop resistance over time that would circumvent the pipeline of antimicrobial drug discovery [64]. In these terms, additional targeting of the *S. aureus* teichoic acid-associated β-glycosyltransferase enzyme (TarS) has been considered beneficial to hamper methicillin resistance [65]. This transferase enzyme has been reported to involve several mechanistic aspects enrolled with *S. aureus’s* ability to cope with microenvironmental stresses, biofilm formation, evasion of immune responses, lysozyme resistances, and triggering inflammatory responses [66,67,68,69,70,71]. The enzyme is chiefly responsible for beta-acylation of bacterial cell wall teichoic acid via *N*-acetylglucosamine being implicated within the IgG-mediated opsonophagocytosis and complement activations at clinical-isolated *S. aureus* strains [72]. Furthermore, resensitization of MRSA strains towards β-lactam antibiotics was reported to be achieved following TarS deletion [73].

For investigating the herein reported anti-human coronavirus 229E (HCoV-229E) activity, targeting the virus main protease (Mpro) has been considered ideal for developing broad-spectrum targeted therapeutics [74]. Owing to the high structural conservation in general across different coronavirus lineages and their integral role within the virus life cycle, these proteases represent promising targets for hampering the virus activities [75,76]. Three coronavirus lineages are identified and classified according to the degree of strain pathogenicity towards humans (lower, HCoV-229E; moderate, HCoV-OC43; and higher, SARS-CoV-2). Thus, targeting singular lineage’s Mpro would harbor the potentiality to target, at least to a great extent, the other coronavirus lineages [77,78,79]. Moreover, these viral proteases lack homologous assemblies within the human biological systems, which highlights the potential safety profile for Mpro inhibitors to any other coronavirus biotargets [80].

Throughout our docking investigation and across the above four designated targets, two identified phytochemicals were depicted with the highest promising affinities (Table 5). Showing high negative docking binding energies (ΔG), both orientin and heptelidic acid were considered promising as multi-target drugs against relevant antibacterial and antiviral target molecules. Docking energies for both phytochemical compounds were higher at *S. aureus* targets MurE and *HCoV-229E* Mpro as compared to their respective ones towards *S. aureus* PBP2a and TarS. On the contrary, both ferulic acid and kojic acid were modest binding energies against all investigated targets conferring their lower relevant affinities for these targets. To our delight, both top-docked phytochemicals depict higher docking scores and predicted affinity towards TarS as compared to a reference target inhibitor. On the other hand, both top-docked compounds had just lower binding energy than the reference inhibitor at the other *S. aureus* targets, MurE and PBP2a, while being only ~0.25-fold lower than the Mpro reference compound.

Reference compounds were adopted throughout the docking investigation to ensure the clinical significance of the docking findings. Applying the same docking protocol and algorithm for the reported target inhibitor and/or relevant co-crystalline ligand serves as positive control references permitting comparative docking findings with reported experimental data [81,82]. Herein, a thiazolidinylidene-based compound (T26) was adopted as a positive control as a MurE inhibitor. The reference compound was reported with high dual inhibition activities towards MurE and MurD from *S. aureus* (IC_50_ = 17.0 μM and 6.4 μM, respectively) [83]. Reported studies highlighted close similarity between the MurE secondary structure originating from MRSA and *E. Coli* microorganisms (RMSD 1.48 Å along > 450 Cα-atoms and Z-score 21.2) [60,84]. Furthermore, T26 highlighted great antibacterial activity against MRSA and its wildtype strain with a minimum inhibition concentration of 9.0 μg/mL [83]. Concerning PBP2a, the co-crystallized cephalosporin antibiotic, ceftaroline [85], was a relevant positive control. The novel 5th generation *β*-lactam drug exhibits broad-spectrum activities, particularly towards the gram-negative bacteria and highly resistant microorganisms, including MRSA, vancomycin-resistant, -intermediate and heteroresistant vancomycin-intermediate *S. aureus* strains [86]. The co-crystallized TarS substrate (UDP-GluNAc) was the suitable comparator, where the ability of investigated phytochemicals to achieve higher docking energies would confer their ability to competitively displace the substrate and hamper the enzyme machinery [65]. Finally, the co-crystallized HCoV-229E Mpro ligand, nirmatrelvir, was adopted as a relevant reference compound where this peptide-like small molecule served as a potent inhibitor of the SARS-CoV2 Mpro enzyme with IC_50_ = 0.79 nM and Ki = 3 nM [87]. The superior docking score of nirmatrelvir can be highly rationalized to its reported great inhibition activity down to the low nanomolar concentrations.

**Figure 8:**
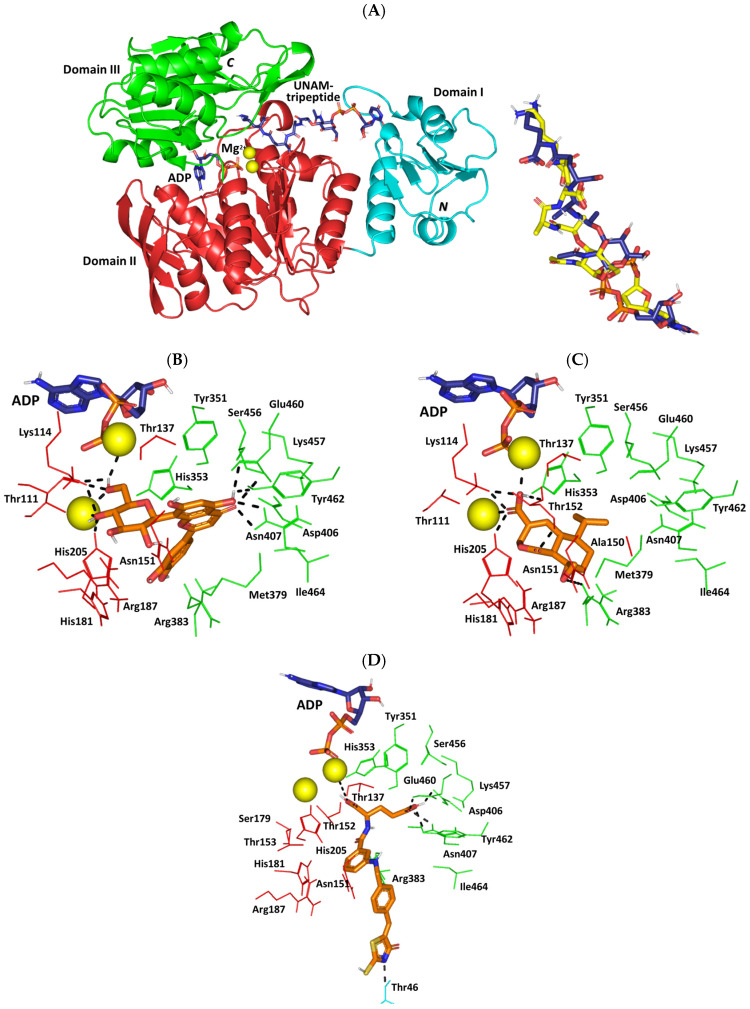
The architecture of *S. aureus* MurE and depicted molecular docking poses. (**A**) **Left panel**: Cartoon 3D-representation of *S. aureus* MurE (PDB; 4c12) ligase showing structural domains; I, II, and III (cyan, red, and green, respectively) bound to two magnesium ions (yellow) and the co-crystallized adenosine diphosphate (ADP) and product UDP-*N*-acetylmuramyl-tripeptide (UNAM-tripeptide) as blue sticks. Bold *C* and *N* letters denote the carboxy and amino terminals. **Right Panel**: Aligned redocked MurE product (UNAM-tripeptide; yellow) over its co-crystalline state (blue). Predicted binding mode of (**B**) orientin, (**C**) heptelidic acid, and (**D**) antibacterial T26 as reference ligand. Only surrounding residues within 5 Å radius as lines are shown and polar interactions are illustrated as black dash lines.

To highlight the differential binding affinities for the top-docked phytochemicals, a comprehensive evaluation of the ligand’s orientation/conformation and residue-wise interactions were undertaken at each target. Interestingly, molecular docking of orientin and heptelidic acid at *S. aureus* MurE revealed preferential anchoring of the hypertensive compound at the binding domain of co-crystallized product UDP-*N*-acetylmuramyl-tripeptide (UNAM-tripeptide). Typically, the product binds predominantly across the central domain of the ligase protein near the ATP-binding site (Figure 8A). Several MRSA MurE key residues have been reported as important, including Asp406, Ser456, and Glu460, for product/substrate binding and recognition [60], as well as an affinity for promising inhibitors [88,89,90,91]. Both the negatively charged sidechains of Asp406 and Glu460 as well as the polar mainchain of Ser456 served as the electrostatic trap mediating the stability of UNAM-tripeptide at the binding site. Validation of the docking protocol was highlighted through redocking the co-crystallized ligand under the same adopted parameter, highlighting great superimposed alignment for the co-crystallized and redocked conformation (RMSD = 1.8 Å) (Figure 8A). Furnishing RMSD below 2.0 Å for the co-crystallized ligand to its reference conformation/orientation signifies that both the adopted docking parameters and algorithms were efficient for predicting relevant binding poses, highlighting respective biological significance and, in turn, the docking energies [92].

Residue-wise interactions for orientin and heptelidic acid depicted a wide polar network with surrounding residues. Orientin predicted polar interactions with magnesium ions, besides hydrogen bonding at (hydrogen bond-Donor–Acceptor at angles/distances) with Tyr462 sidechain –OH (2.8Å/135.2°), Thr111 mainchain C=O (2.1Å/129.1°), Lys114 sidechain –N^+^H_3_ (2.5Å/128.2° and 2.1Å/144.2°), His205 sidechain NHτ (2.3Å/124.2°), Asn407 sidechain –NH_2_ (2.4Å/126.7° and 2.3Å/121.1°), and Glu460 sidechain OH (2.4Å/128.0°). Furthermore, several hydrophobic contacts with surrounding non-polar residues are also shown in Figure 8B. Hydrophobic π-mediated contacts with Tyr351 further stabilized orientin at the catalytic site. Owing to its smaller size, heptelidic acid predicted a preferential orientation towards domain II of the active site furnishing several polar contacts with Mg^2+^, Lys114 sidechain –N^+^H_3_ (2.1Å/155.7° and 2.7Å/133.8°), Thr152 (sidechain OH; 2.3Å/174.1° and mainchain –NH; 3.0Å/144.2°), His205 sidechain NHτ (2.5Å/152.6°), and Arg383 sidechain–NHτ– (3.1Å/124.8°). The latter could rationalize the inferior docking score of heptelidic acid as compared to orientin. The sesquiterpene lactone phytochemical predicted favored van der Waal contacts via its hydrophobic cage-like structure with the surrounding pocket residues including Ala150, His181, His353, and Met379 (Figure 8C). Finally, docking of the reference positive control, T26, at MurE highlighted dominant electrostatic potentiality guiding its anchoring at the substrate site with extended orientation/conformation across the domain I/II/III of the active site explaining its relatively higher docking energy as compared to docked phytochemicals. Interactions with Mg^2+^, Thr46 sidechain OH (2.6 Å/159.1°), Asp406 sidechain C=O (2.6 Å/123.3°), Asn407 sidechain –NH_2_ (2.6 Å/118.4° and 2.8 Å/115.0°), and Glu460 sidechain –OH (2.0 Å/140.5°) residues were highlighted (Figure 8D).

**Figure 9:**
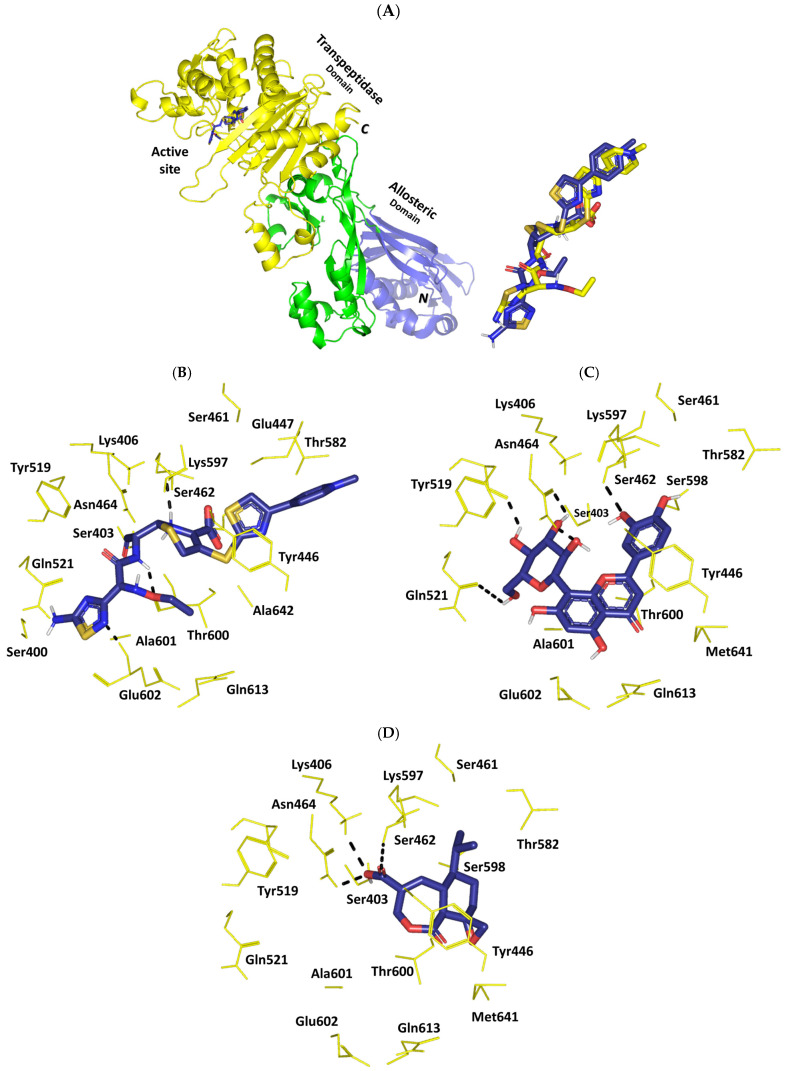
The architecture of *S. aureus* PBP2a and depicted molecular docking poses. (**A**) **Left Panel**: Cartoon 3D-representation of *S. aureus* PBP2a (PDB; 3zg0) transpeptidase enzyme in complex with ceftaroline co-crystalline ligand (blue sticks) and showing structural domains; transpeptidase domain (yellow) and allosteric domain (blue green). Bold letters *C* and *N* denote carboxy and amino terminals. **Right Panel**: Aligned redocked ceftaroline (yellow) over its co-crystalline state (blue); predicted binding mode of (**B**) ceftaroline as positive reference control, (**C**) orientin, and (**D**) heptelidic acid. Only surrounding residues within a 5 Å radius as lines are shown and colored as per constituting domains. Polar interactions are illustrated as black dash lines.

Exploring the final stage of peptidoglycan synthesis, targeting PBP2a has been considered beneficial for hampering MRSA survival. Generally, the catalytic active site of PBP2a resides at the transpeptidase domain residing within an open groove on the protein surface readily accessible to ligands (Figure 9A) [85]. Notably, three conserved motifs have been reported to cluster around the active sites while harboring the catalytic serine and all the residues required to activate the catalytic hydroxyl group for a nucleophilic attack. The first motif comprises the S-X-X-K (Ser403-Thr404-Gln405-Lys406) tetrad where the catalytic serine resides and its sidekick, lysine amino acid, can exhibit their vital role for organizing the nearby residues as well as minimizing the pKa of the catalytic serine-OH [93]. The second and third conserved motifs are composed of the S-X-N (Ser462-Asp463-Asn464) and K-X-G (Lys570-Ser571-Gly572) triads. The characteristic tetrad and triad motifs adopt strikingly similar conformations in a way that makes all active sites within the serine-based PBPs appear just the same [94]. Interestingly, the β-lactam-inhibiting enzymes (β-lactamases), which are responsible for bacteria resistance through β-lactam catalytic hydrolysis, exhibit the same three conserved motifs making them evolutionary and mechanistically related to all PBPs [94,95]. Such observations explained how penicillins, cephalosporins, and carbapenems exhibit affinity for several PBPs and β-lactamases, where the latter can confer bacterial resistance. Therefore, introducing non-β-lactam-based antimicrobial agents, like propranolol, to circumvent the overgrowing resistance against β-lactam antibiotics is considered highly rationalized [95].

Redocking the co-crystallized cephalosporin antibiotic, ceftaroline [85], provided a validation tool for the adopted docking protocol and algorithm. At the depicted aligned RMSD of 0.5 Å, the redocked ceftaroline managed to replicate its co-crystallized conformation/orientation and residue-wise patterns (Figure 9A). Polar interaction with Ser462 sidechain –OH (2.2 Å/157.4°), Thr600 mainchain C=O (2.1 Å/169.6°), and Glu602 sidechain –NH (1.8 Å/140.3°) were conserved towards the ligand’s polar functionalities of the opened β-lactam ring, amidic sidechain, and thiadiazole ring substitution (Figure 9B). Stacking between the ligand’s thiazole ring and Tyr446 sidechain through close range π-π hydrophobic contact (4.1 Å) provides extra stability near the conserved S-X-N motif. Docking orientin at PBP2a was dominant through polar interaction with the Tyr519 mainchain C=O (2.1 Å/133.2°), Gln521 sidechain C=O (2.9 Å/127.4°), Ser462 sidechain –OH (2.4 Å/121.0°), and Asn464 sidechain (–NH_2_; 2.8 Å/162.4° and C=O; 2.6 Å/124.7°). Orientin stability was further mediated through non-polar contacts with surrounding residues (Ala601 and Met641) as well as hydrophobic π–π interaction between the compound’s resorcinol ring and the Tyr446 sidechain (Figure 9C). For heptelidic acid, limited polar interactions were depicted at the PBP2a binding site since few polar networks were seen with the Lys406 sidechain –N^+^H_3_ (2.8 Å/162.4°), Ser462 sidechain –OH (2.8 Å/162.4°), and Asn464 sidechain –NH_2_ (2.8 Å/162.4°) (Figure 9D). The latter docking observation could be related to less inherited structural flexibility of heptelidic acid, the thing that limits its conformational maneuvers conferring a lower docking score to orientin. The lack of the compound’s aromaticity could provide a reason for the fewer hydrophobic interactions depicted by heptelidic acid towards the pocket lining residues.

**Figure 10:**
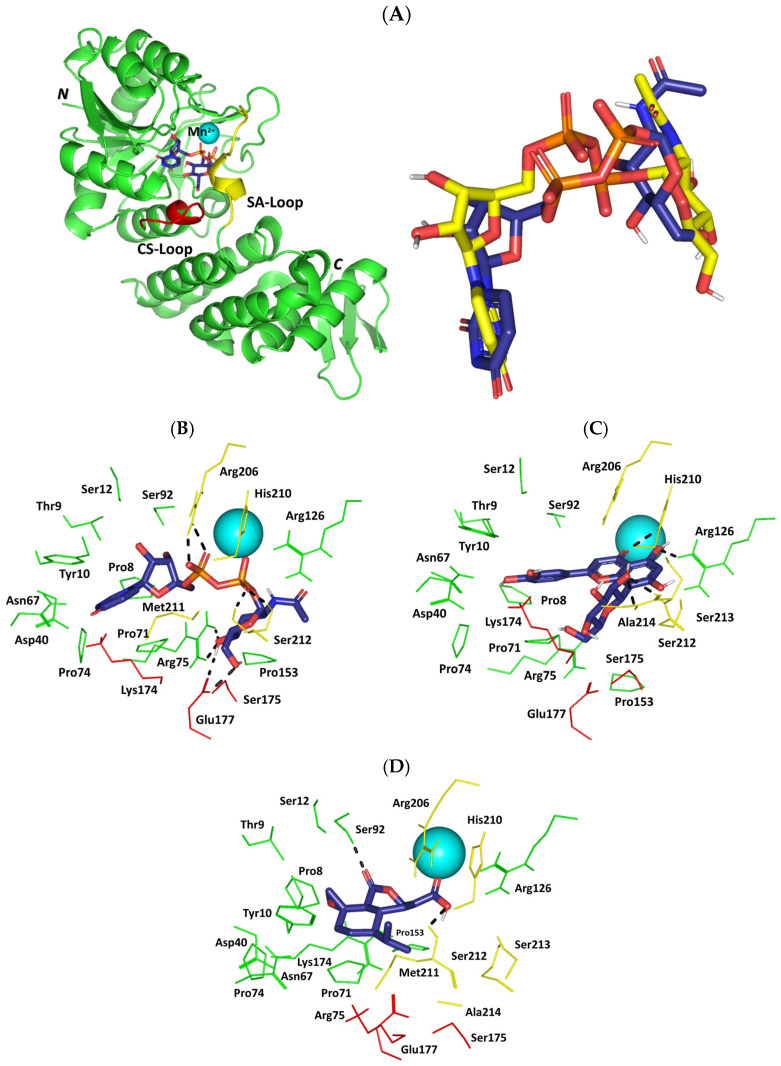
The architecture of *S. aureus* TarS and depicted molecular docking poses. (**A**) **Left Panel**: Cartoon 3D-representation of *S. aureus* TarS (PDB; 5tzj) catalytic domain in complex with co-crystalline substrate (UDP-GluNAc; blue sticks) with key structural loops; CS-loop (Glu171–Asp178; red) and SA-loop (Lys205–Tyr215; yellow). Bold letters *C* and *N* denote carboxy and amino terminals. **Right Panel**: Aligned redocked UDP-GluNAc (yellow) over its co-crystalline state (blue). Predicted binding mode of (**B**) UDP-GluNAc as a positive reference control, (**C**) orientin, and (**D**) heptelidic acid. Only surrounding residues within a 5 Å radius as lines are shown and colored as per constituting domains. Polar interactions are illustrated as black dash lines.

Investigating the compounds’ residue-wise interactions at the TarS catalytic site would provide valuable insights regarding the ability of top-docked phytochemicals to interfere with bacterial virulence and biofilm production [66,67,68,69,70,71]. The enzyme catalytic site is settled at the carboxy-terminal domain exhibiting the canonical GTA folding (double α/β/α sandwiched Rossman motifs) (Figure 10A) [65]. The binding site is enclosed within two key loops: (a) the catalytic site loop (CS-loop; Glu171–Asp178) encompassing the putative base catalytic residue Asp178; (b) the substrate access loop (SA-loop; Lys205–Tyr215). Several pocket residues including Tyr10, Arg75, Asp91, Glu177, Asp178, His210, and Ser212 have been reported as important for recognizing and binding the enzyme’s substrate (Uridine diphosphate *N*-acetylglucosamine; UDP-GluNAc) as well as small molecule TarS inhibitors [65,96,97,98]. Preliminary redocking of the co-crystallized substrate revealed the validity of the adopted docking protocol where UDP-GluNAc achieved low RMSD (0.9 Å) to its co-crystalline orientation/conformation (Figure 10A). The redocked substrate recaptured the co-crystallized residue-wise interaction patterns including salt bridges with vicinal residues including Arg75 sidechain (sidechain =N^+^H_2_; 1.9 Å/150.1° and sidechain –N^+^H_2_; 2.3 Å/134.7°), Glu177 sidechain (C=O; 1.8 Å/148.0° and –O^−^; 3.0 Å/128.8°), Arg206 sidechain –N^+^H_2_ (2.2 Å/123.6° and 2.5 Å/124.7°), and Ser212 (mainchain –NH; 1.9 Å/164.0° and sidechain –OH; 2.7 Å/127.3°). Hydrophobic contacts with several prolines (Pro8, Pro71, Pro74, and Pro153) as well as π–π stacking for the pyrimidindione ring with Tyr10 were also relevant at close proximities (Figure 10B). Docking interactions for the identified top-docked phytochemicals were mostly differentiated based on polar contacts with surrounding residues. Owing to the higher number of hydrogen bond donors and acceptors for orientin as compared to heptelidic acid, the earlier depicted a wider polar network towards pocket-lining residues. Hydrogen bonds with Arg126 sidechain =N^+^H_2_ (2.3 Å/129.4°), His210 sidechain NHτ (2.9 Å/125.5°), Ser213 mainchain –NH (2.3 Å/142.9°), and Ala214 mainchain –NH (2.6 Å/123.9°) were predicted for orientin at optimum angles and distances (Figure 10C). Displaced face-to-face π–π stacking was depicted between orientin’s resorcinol ring and Tyr10 at a close distance. Fewer polar contacts were depicted for heptelidic acid (Ser92 sidechain –OH; 2.0 Å/152.7° and Met211 mainchain –NH; 2.3 Å/124.7°) with limited hydrophobic contacts (Figure 10D).

**Figure 11:**
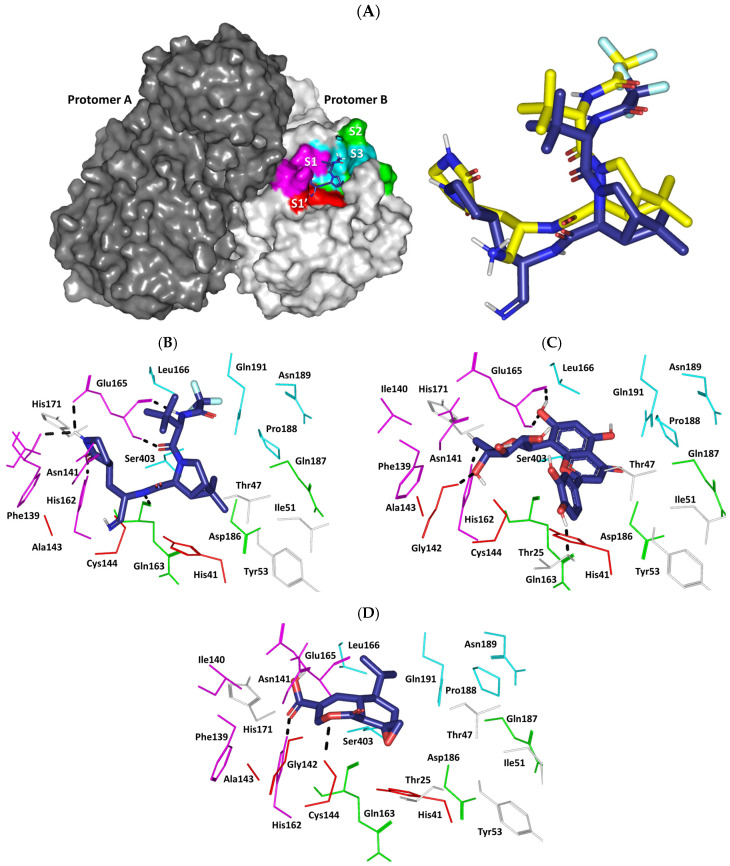
The architecture of HCoV-229E Mpro and depicted molecular docking poses. (**A**) **Left Panel**: Cartoon 3D-representation of HCoV-229E Mpro (PDB; 7yrz) in its dimeric state (dark/light grey surface colors for respective protomers A/B) with its canonical substrate binding site comprising important sub-sites (S1′ as red, S1 as magenta, S2 as green, and S3 as cyan). **Right Panel**: Aligned redocked nirmatrelvir (yellow) over its co-crystalline state (blue); predicted binding mode of (**B**) Nirmatrelvir as positive reference control, (**C**) orientin, and (**D**) heptelidic acid. Only surrounding residues within a 5 Å radius as lines are shown and colored as per constituting domains. Polar interactions are illustrated as black dash lines.

Moving towards the compound’s differential Mpro-related affinities, a comprehensive examination of the ligand’s residue-wise interaction was conducted. Typically, the target’s substrate pocket illustrated that the co-crystallized binary complex comprises four sub-sites (S_1_′-to-S_4_) for anchoring the four peptido-partitions (P_1_′-to-P_4_) of its substrate (Figure 11A) [74]. Within the current literature, several Mpro pocket amino acids are identified as important for binding different ligands [77,80,99,100,101]. Binding to the S1′ sub-site, especially towards the Mpro catalytic dyads His41 and Cys144, has been identified as important for strong ligand–protein interactions and enzyme hydrolytic activity blockage [102]. Significant non-polar contacts with the sidechains of either Glu165 or Asn189 at the S3 sub-site, as well as S2 sub-site residues (Ala49 and Leu190), can serve as hydrophobic grips for anchoring different small molecules in the enzyme’s pocket [74]. Regarding polar binding interactions, both carbonyl and nitrogen of the Glu165 mainchain were highlighted as crucial for providing relevant ligand–Mpro binding at the S1 sub-site. Several other amino acids including Asn24, Thr25, Ser168, His171, Phe184, and Ala195 were reported in literature as being relevant for preferential ligand binding [99,100,101]. Initially, the furnished docking poses and energies were considered valid since preliminary redocking of co-crystallized nirmatrelvir showed a root-mean-squared deviation: RMSD = 1.72 Å (Figure 11A). Redocked nirmatrelvir was able to replicate the co-crystallized ligand–Mpro binding interactions showing double polar hydrogen bonds with the S1 pocket Glu165 mainchain (–NH; 1.9 Å/171.8° and C=O; 2.0 Å/168.9°) via the ligand’s amide moiety (Figure 11B). The ligand’s pyrrolidinyl moiety mediated polar interaction with the Glu165 sidechain oxyanion (2.5 Å/128.2°) and the Phe139 mainchain C=O (2.5 Å/141.6°). The ring further mediated the hydrogen bond with the S1 pocket His162 sidechain NHτ (1.8 Å/168.4°). Additional hydrogen bonds between the compound’s central amide linker and the S2 pocket Gln163 mainchain C=O (2.2 Å/163.8°) were also depicted. Close-range hydrophobic interactions (π-CH) towards His41 and the ligand’s bicyclic ring (5.0 Å) served to further the ligand’s stability at the S1′ sub-site. Further van der Waal contacts with non-polar pocket lining residues Ile51, Ala143, Ile164, Leu166, and Pro188 were observed. Altogether, these favored ligand–target interactions would be translated into superior docking binding scores corresponding to the reported experimental in vitro Mpro inhibition assay (IC_50_ at low-range nanomolar concentration). To our delight, the identified phytochemicals revealed relevant ligand accommodation at the Mpro binding site. Orientin depicted extended orientation across the four sub-sites, S1′–S3 (Figure 11C). Lodging at the S1′ sub-site was solely relevant for the orientin as compared to the other identified phytochemicals through polar interaction with Gly142 mainchain –NH (2.2 Å/163.8°). Further T-shaped π–π hydrophobic contact was shown between the compound’s resorcinol ring and S1′s pocket His41 sidechain. Further polar interactions were predicted for orientin including residues of pockets S1 Glu165 mainchain (–NH; 2.2 Å/163.8° and C=O; 2.1 Å/146.5°), His162 sidechain NHτ (2.2 Å/163.8°), as well as vicinal residue Thr25 sidechain –OH (2.2 Å/163.8°). Non-polar van der Waal contacts with Ile51, Phe139, Ile140, Ala143, and Pro188 were also depicted. Moving to heptelidic acid, a lower extent of polar interaction was depicted. Interactions with Pocket S1 His162 sidechain NHτ (2.2 Å/156.4°) and S1′ Cys144 sidechain –SH (2.9 Å/140.5°) were only depicted for the sesquiterpene lactone derivative (Figure 11D). Such differential ligand’s residue-wise interactions confer higher docking energy for orientin regarding heptelidic acid. Despite limited polar interactions, heptelidic acid is predicted to mediate several non-polar contacts with several residues (His41, Ile164, Leu166, and Pro188) owing to its cage-like architecture and isopropyl arm chain. This could partially compensate for the limited electrostatic interactions predicted by this smaller-sized phytochemical compound.

### 2.9. Molecular Dynamics Studies

**Figure 12:**
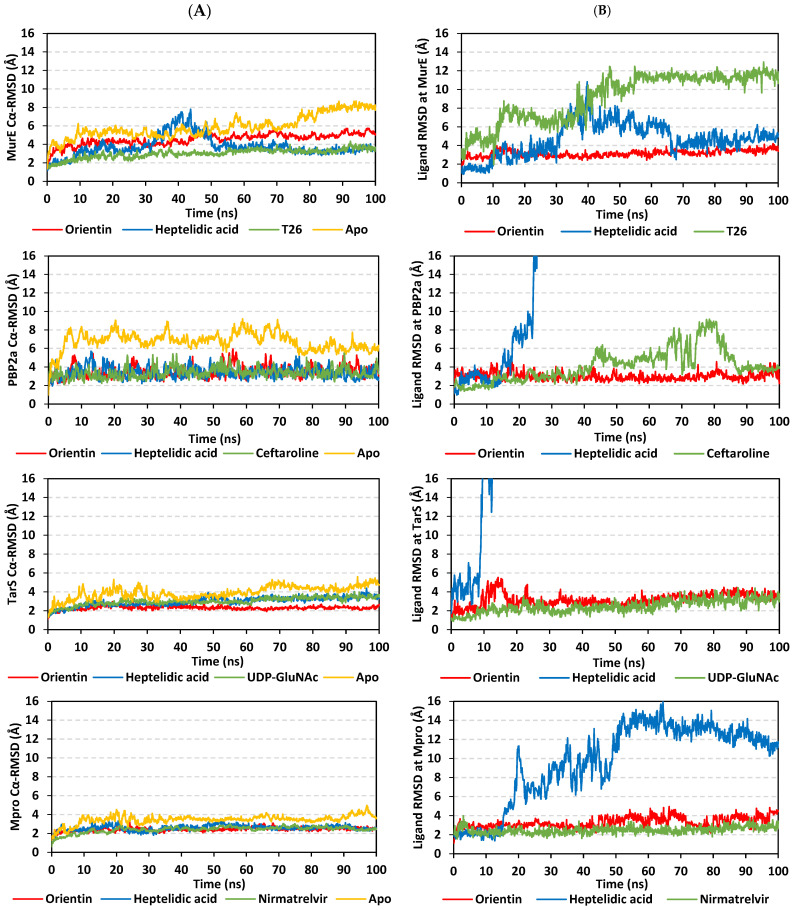
Thermodynamic stability analysis of the explicit molecular dynamics simulated compounds inbound to *S. aureus* and HCoV-229E biotargets. (**A**) Alpha-carbon atom RMSDs for protein (holo and apo states); (**B**) sole ligand RMSDs, in relation to simulation timeframes (ns).

Thermodynamic behaviors of the identified compound–target proteins complexes were monitored to positive reference compounds (MurE: T26, PBP2a: Ceftaroline, TarS: UDP-GluNAc, and Mpro: Nirmatrelvir) as well as the Apo protein states through explicit molecular dynamics simulation. This approach has provided valuable molecular insights regarding compound–target relative stabilities, conformational changes, and favored interactions under near-physiological conditions [103,104,105]. Regarding the initial structures, the RMSD trajectories were tracked for the simulated bound proteins. Altered conformations and compromised stability are typically correlated with high target RMSDs [106], whereas ligands with excellent pocket accommodation are related to steady and small-value RMSDs [107]. Simulated proteins showed typical thermodynamic behaviors as alpha-carbon RMSD trajectories showed low initial values that increased within the first few steps and then more or less leveled off around respective averages for more than half of the simulations (Figure 12A). Interestingly, monitored RMSDs for all compound-bound (holo) proteins were at lower average values and less fluctuating tones (4.54 ± 0.61 Å to 3.00 ± 0.49 Å for MurE, 3.40 ± 0.57 Å to 3.47 ± 0.63 Å for PBP2a, 2.30 ± 0.20 Å to 3.00 ± 0.41 Å for TarS, and 2.46 ± 0.33 Å to 2.58 ± 0.28 Å for Mpro) as compared to the apo state (without bound compound/unliganded) (5.92 ± 1.22 Å for MurE, 6.77 ± 0.96 Å for PBP2a, 3.92 ± 0.67 Å for TarS, and 3.46 ± 0.47 Å for Mpro). Higher fluctuation patterns (in terms of magnitudes and/or frequencies) were assigned for proteins inbound with heptelidic acid over those of orientin only at TarS, while being indistinguishable across the PBP2a and Mpro simulations. In terms of MurE protein RMSDs, the heptelidic acid-bound protein exhibited higher tones than that of orientin only across 30–70 ns, while kept at lower values for most of the simulation run. Differential RMSDs for simulated proteins showed higher fluctuations for MurE and PBP2a as compared to those of TarS and Mpro. These depicted dynamic behaviors can be partially correlated to the differential secondary structure and B-factor index of the bound proteins.

**Figure 13:**
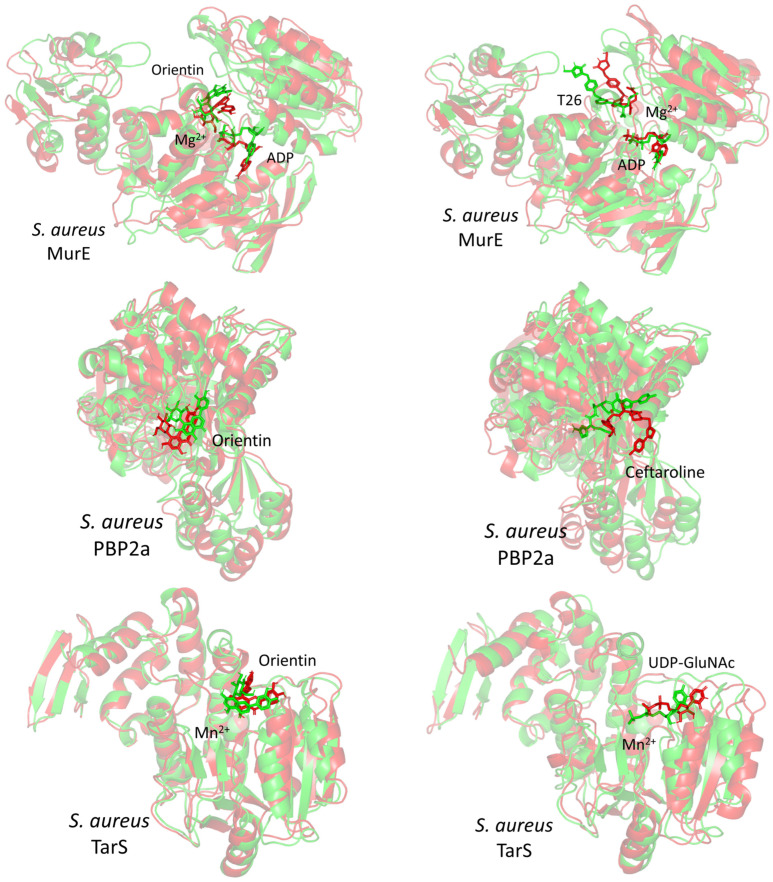

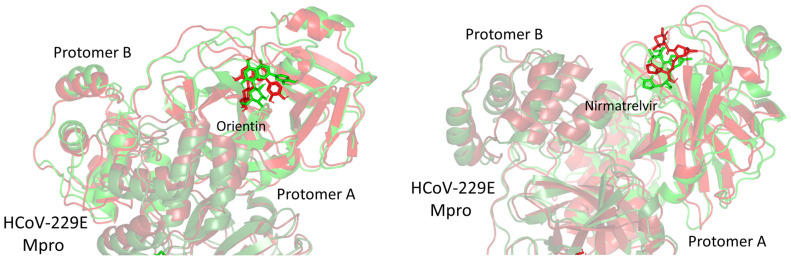
Conformational analysis for molecular dynamics simulated compounds inbound to *S. aureus* and HCoV-229E biotargets. Overlaid ligand–target snapshots at initial and final timeframes. Top-stable compounds (orientin and reference ligands—sticks) and bound proteins (cartoons) are colored green and red concerning 0 ns and 100 ns extracted frames.

Concerning the sole simulated compound monitoring, the ligands’ RMSDs were monitored through selecting the carbon-alpha center of their respective bound proteins for their least-square fit analysis. The grouped ligands atoms (based on the GROMACS index.file) were selected to perform the RMSD analysis. Applying such approaches have provided a relevant description to understand whether a specific ligand was retained within its binding pose, confined within the binding site, or not throughout the dynamics runs. Notably, RMSDs of respective compounds highlighted differential stability across the simulated times (Figure 12B). As a general observation, limited fluctuations with steady RMSD tones were assigned for orientin across all bound protein systems (3.25 ± 0.50 Å for MurE, 3.01 ± 0.42 Å for PBP2a, 3.17 ± 0.68 Å for TarS, and 3.29 ± 0.56 Å for Mpro) as compared to heptelidic acid (4.68 ± 1.78 Å for MurE, 64.43 ± 39.37 Å for PBP2a, 35.29 ± 11.97 Å for TarS, and 9.67 ± 4.09 Å for Mpro). The orientin’s RMSD tones within the MurE complex were observed statistically indistinguishable from its own ones at the other three complexes. On the other hand, orientin’s RMSD trajectories were depicted comparable to those of the TarS reference ligand (UDP-GluNAc; 2.48 ± 0.68 Å) and positive controls at the Mpro model (Nirmatrelvir; 2.48 ± 0.36 Å), yet even lower than references at the MurE and PBP2a models (T26; 9.17 ± 2.57 Å and Ceftaroline; 4.16 ± 1.74 Å, respectively). The latter thermodynamic behaviors would confer preferential orientin’s dynamic stability and confinement within the different target binding sites. Despite higher fluctuations for heptelidic acid, its RMSDs leveled off around an average value starting from a 55-to-60 ns timeframe and till the end of the simulation runs only at the MurE and Mpro systems. Notably, T26 at the MurE complex across 30–70 ns showed high RMSDs before they descend and come to their initial tones. In cases of PBP2a and TarS complexes, heptelidic acid RMSDs were far beyond range (>16 Å) conferring significant drift at new protein sites much farther away from the initial location at the proteins’ canonical binding site. Both large RMSDs for heptelidic acid in all complexes and T26 in the MurE complex confer that the ligands moved from their original binding sites to new ones but still remained in contact with the protein, rather than simply dissociating into the solvent. For T26 at MurE, the ligand’s drift was quite transit as it managed to return back to the initial site for the last 30 ns of the simulation run. It is worth noting that the depicted RMSDs for orientin and reference ligands never exceeded 2.5-fold the RMSD values of their respective bound proteins, with an exception only for T26 at MurE (~ 4.0-folds). This has been confirmed relevant in the literature for the compound’s existence within the binding site as well as successful protein convergence at the end of the simulation demanding no further time extensions [108,109]. Further compound-active site stability was investigated through the time evolution of the ligand–protein complex conformations and ligand orientation analysis. Overlaid timeframes at the beginning and end of the simulation run confirm orientin and reference ligand as relevant accommodations of the binding site (Figure 13).

**Figure 14:**
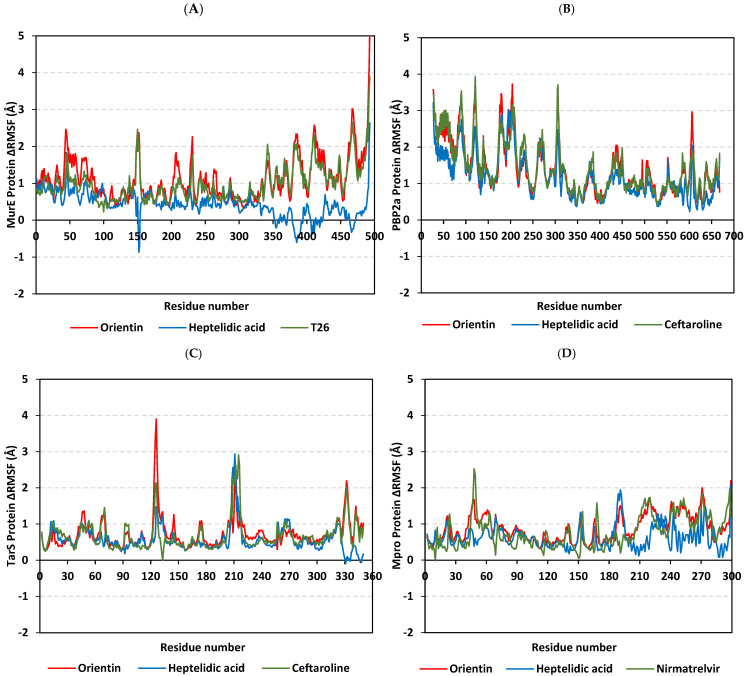
Global stability of simulated proteins down to their constituting residues. Difference RMSF analysis of inbound *S. aureus* (**A**) MurE, (**B**) PBP2a, (**C**) TarS, and (**D**) HCoV-229E Mpro proteins along the whole molecular dynamics runs highlighting the residue-wise flexible contributions of holoprotein in relation to the apo/unliganded states.

Monitoring the RMS fluctuations for the holo/apo target proteins to their alpha-carbon references provided further stability analysis. Protein stability and flexibility/immobility profiles were dissected down to their constituent amino acids [110]. RMSFs allow the researchers to comprehend the residue-wise dynamic behaviors at the protein’s binding pocket/vicinal loops in addition to pinpointing the key amino acids being significant for ligand binding [111,112]. Normalized RMSFs (ΔRMSF = apoRMSF − holoRMSF) were adopted as better representations of the protein’s local flexibility in relation to its apo state. Adopting a ΔRMSF cut-off value of 0.30 Å has been reported as relevant for estimating the significant alterations within the protein’s structural movements, meaning that residues depicting ΔRMSF greater than 0.30 Å indicated reduced backbone mobility upon binding [113]. In concordance with the RMSD findings, lower flexibility and mobility tones across almost all protein regions were assigned for the holo proteins in relation to their apo states where the earlier were shown with almost positive ΔRMSF values (Figure 14). This confers the impact of ligand binding on the stabilizing of target proteins’ secondary structures. This further suggests that ligand binding would impact protein stability in a manner much extended beyond the canonical binding site affecting even the far protein regions. Additionally, typical protein dynamic behavior was illustrated since higher flexibility profiles were seen for the terminal residues as compared to the core regions, except for the carboxy terminals of *S. aureus* MurE proteins bound with orientin and T26 where binding sites are at proximity distances to the protein’s C-terminus. Secondly, the stability-driven impacts of orientin and reference compound binding on the four protein targets were more profound than those of heptelidic acid where the latter was assigned with lower ΔRMSF values. This would further highlight the lower stability profiles of heptelidic acid–protein complexes in relation to orientin and reference compounds in good agreement with ligand drift away from the initial binding site.

**Figure 15:**
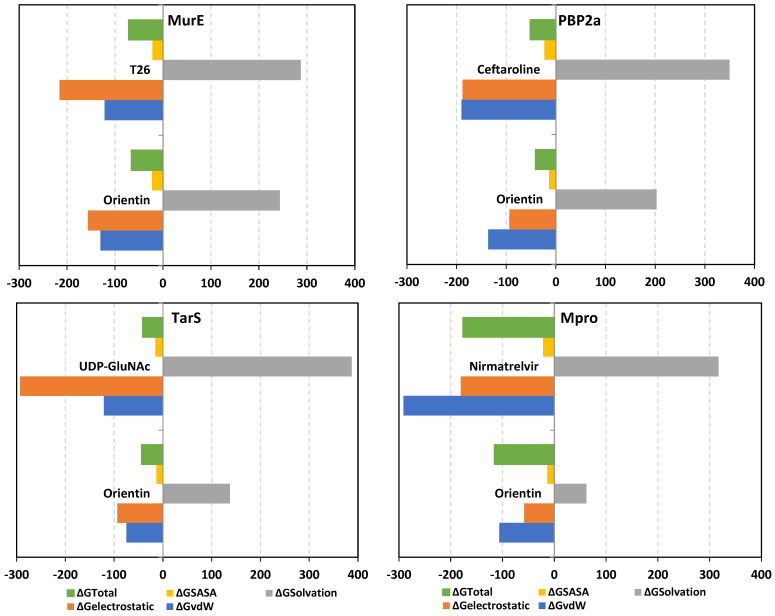
MM_PBSA free-binding energy calculations and constituting energy term contributions for the ligand–protein target complexes.

Free-binding energy calculations using the trajectory-oriented Molecular Mechanics-Poisson Boltzmann Surface Area (MM-PBSA) approach were performed to understand the nature of top-stable ligand–protein binding, estimating affinity magnitude, as well as individual energy contribution of key binding residues [114]. MM-PBSA calculation possesses the advantage of being comparably accurate to free-energy perturbations, yet with lower computational expenditure [115]. Notably, the free binding energies of the simulated orientin were quite second to the simulated reference compounds at the complex targets: MurE (−66.00 ± 4.46 vs. −71.70 ± 13.08), PBP2a (−41.09 ± 6.87 vs. −51.66 ± 35.89), and Mpro (−115.41 ± 14.87 vs. −176.27 ± 16.42), except for TarS where the identified phytochemical was just superior (−43.76 ± 12.58 vs. −41.51 ± 46.35) (Figure 15). However, the uncertainties on the free binding energies for the reference compounds are so large that they encompass the orientin values. The latter would argue that orientin is second to the reference compound only at the Mpro complex. On the other hand, the provided total ΔG is a relevant translation for all previously presented data including the RMSD and ΔRMSF fluctuations, as well as ligand–target conformational analysis. Just because the Mpro–orientin complex was the one showing the largest difference in total energy from its reference compound does not confer that other protein systems are of negligible difference. In this regard, we would argue that the differential binding free energies for orientin and references incorporate contributions from RMSD and ΔRMSF so that these are already accounted for, due to the fact that the orientin’s binding to a specific protein is quite different as compared to that of the reference compounds. To further confirm such an argument, the differential binding energy terms between orientin and reference compounds were investigated within the forthcoming text. 

Dissection of the free binding energies showed that the electrostatic potential energies (ΔG_electrostatic_) were dominant over van der Waal hydrophobic energy contributions (ΔG_vdW_) driving both the orientin and T26 stabilities at the MurE and TarS complex systems. On the other hand, ΔG_vdW_ showed predominant free-binding energy contributions for orientin’s affinity towards the PBP2a and Mpro models. Dominant ΔG_vdW_ contribution fashions were also depicted with reference ligands only at the PBP2a and Mpro systems, while a profound ΔG_electrostatic_ contribution was seen for UDP-GluNAc at TarS. Interestingly, the high combined non-polar free binding interactions (sum of ΔG_vdW_ and non-polar solvation; ΔG_SASA_) for the simulated ligand–target complexes might be directly related to the targets’ large pocket surface area.

Concerning the polar solvation energy contributions, orientin was assigned with much lower polar solvation energies (ΔG_polar solvation_) across all simulated systems when being compared to reference ligands at corresponding target proteins. The latter was suggested to be in favor of orientin–target affinity since binding has been considered a solvent substitution process [116,117,118,119,120]. Harboring significant aromatic/heterocyclic structural features could allow reasonable compensation of solvation entropy and final relevant total free-binding energy profiles for orientin. On the other hand, higher solvation penalties for reference compounds could be related to the presence of several ionizable groups in contact with hydrophobic pocket sides that would compromise the totally free binding process. Based on the presented structural postulations, prospective structural optimization of orientin can be achieved through balanced hydrophobic/hydrophilic characters. Introducing ionizable scaffolds furnishing increased polarity while possessing relevant aromatic characteristics would be advantageous for minimizing the solvation penalty and maximizing the target affinity. Suggested scaffolds include tetrazole rings and other relevant cyclic carboxylate-related bioisosteres [121].

Finally, it is worth mentioning that significant differential patterns have been highlighted with distinctive energy term preferentiality between the reference compound and orientin at every target system. Thus, from the obtained MM-PBSA calculations, orientin’s binding to a specific protein is quite different as compared to that of the reference compounds, the thing that again successfully reflects the findings obtained at the previous MD analysis parameters, including the RMSD and ΔRMSF analysis as well as the ligand–target conformational investigation.

**Figure 16:**
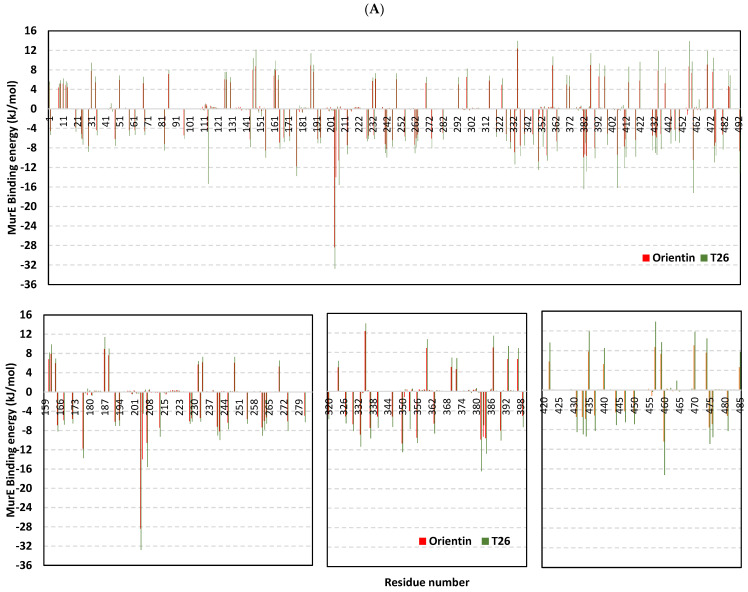

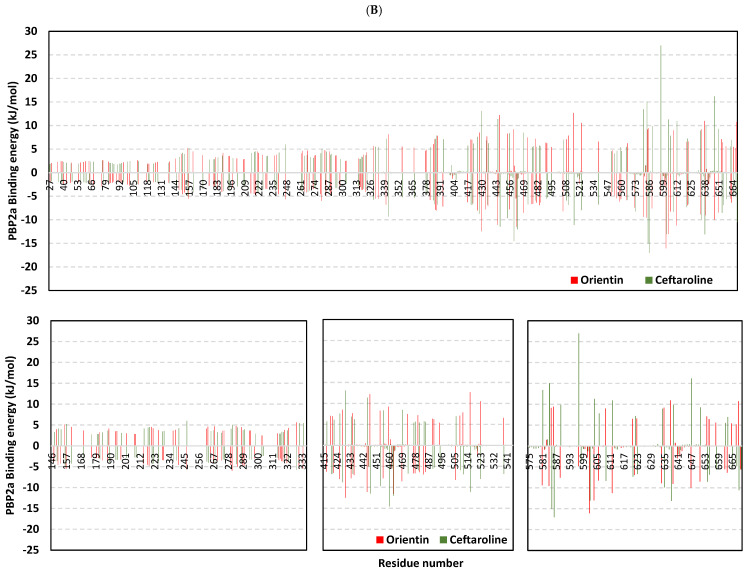

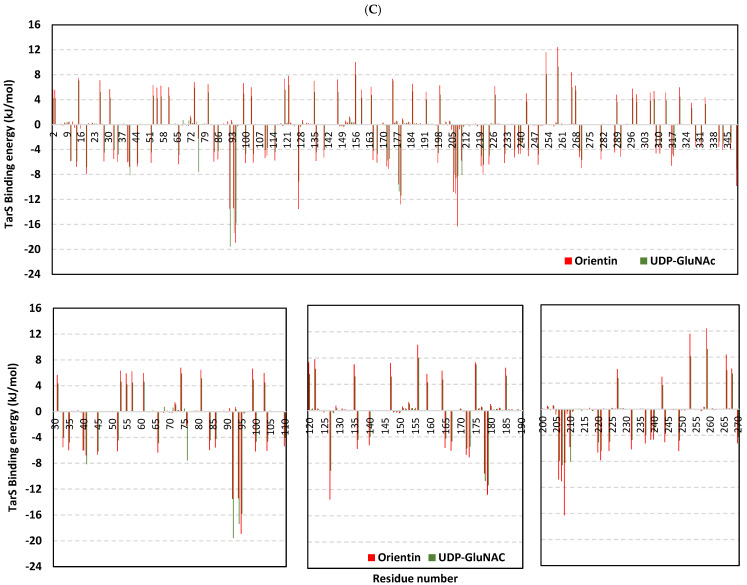

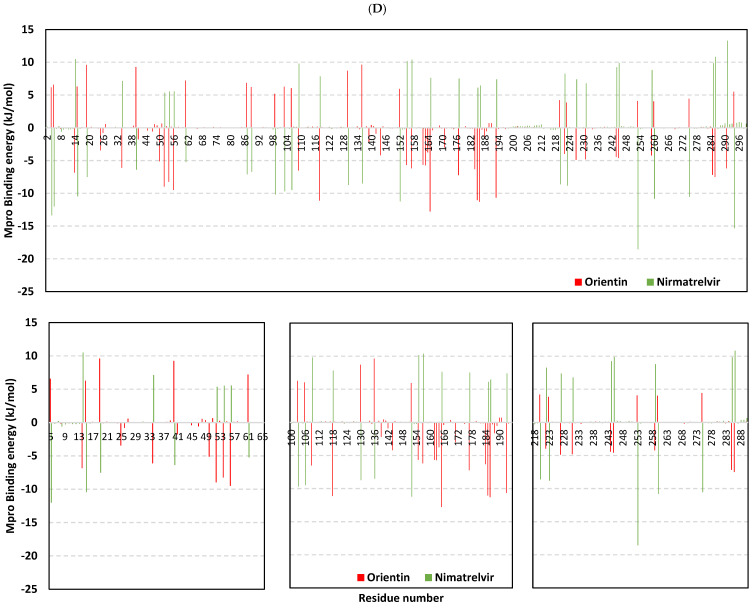
Residue-based energy contributions for ligand–protein target complexes. Lower panels are expanded residue ranges of the simulated protein complexes: (**A**) MurE, (**B**) PBP2a, (**C**) TarS, and (**D**) Mpro, showing their energy contributions within the MM_PBSA free-binding energy calculation.

For gaining more insights concerning ligand–residues interactions, the binding-free energy was dissected down to its residues’ contribution to identifying key residues [115]. Residues of the active binding site depicted favored contributions (large negative values) within the ligand–protein binding energies of orientin and reference ligands (Figure 16). Adopting ≤−5.00 kJ/mol cut-off for significant energy contributions [122], residues Lys62, Lys114, His205, His353, Arg383, Asp406, and Glu460 were illustrated as most important for compound binding at *S. aureus* MurE with the highest contributions being assigned for Lys114, Asp406, Glu460 (−15.37 to −17.24 kJ/mol), and His205 being the most (up to −28.35 kJ/mol). Concerning the PBP2a complex systems, top-favored contributing residues included Tyr446, Ser462, Asp463, Asp573, and Glu602 with the highest contributions for Asp463 (up to −12.03 kJ/mol) and Glu602 (up to −16.08 kJ/mol). Moving to the TarS models, residues like Tyr10, Arg75, Asp91, Asp94, Asp95, Arg126, Glu171, Glu172, Glu177, Asp178, Lys205, Arg206, Glu207, and Glu209 were significant for orientin and UDP-GluNAc binding stability. The dominant polar nature of top-contributing TarS residues further confirms the dominant impact of ΔG_electrostatic_ potentials on ligand binding. For the final target, HCoV-229E Mpro, several residues of the four sub-pocket and vicinal regions were involved in high-negative energy contributions (≤−5.00 kJ/mol), including Ala49, Phe139, Cys144, His162, Gln163, Glu165, His171, and Phe184 with a dominant hydrophobic nature. It is worth mentioning that several other pocket residues showed significant positive energy contributions inferring repulsion forces and unfavored impact on the ligand’s stability. Thus, the addition of balanced hydrophobic/ionizable scaffolds was further highlighted as significant for ligand anchoring. Finally, the above-depicted energy residue-wise findings were consistent with the above-described docking hydrophobic/polar contact preferentiality.

## 3. Materials and Methods

### 3.1. Plant Material

In September 2021, fruits of *Annona squamosa* were acquired from a local Egyptian market. The plant sample was verified by Mrs. Therez Labib, a consultant in plant taxonomy for the Ministry of Agriculture and a former director of the El-Orman Botanical Garden. The Department of Pharmacognosy and Medicinal Plants at Future University’s Faculty of Pharmacy (FUE) received a voucher specimen of the plant material (AS 101).

### 3.2. Isolation of the Endophytic Fungi

Endophytic fungi were separated using the procedure that Fathallah et al. and Hazalin et al. outlined [1,123]. In summary, *A. squamosa* fruits were sterilized for one minute using 70% ethanol, then rinsed twice with sterile water. To prevent bacterial development, the dried fruits (shade drying) were crushed and aseptically added to Potato Dextrose Agar (PDA) (Oxoid, Hampshire, UK) plates supplemented with 250 mg/L of gentamicin and streptomycin. In addition to non-inoculated PDA plates acting as a negative control, non-crushed, surface-sterilized fruits were also grown to rule out the presence of epiphytic fungus. The plates were incubated at 25 °C for seven to fourteen days. Different mycelia that emerged from the segments were grown, and PDA slants were used to preserve the isolated pure fungi.

### 3.3. Morphology of Fungi and Microscopic Analysis

As illustrated by [124], colony characteristics like texture, shape, and color as well as the conventional taxonomic key of the isolated fungus was morphologically identified. A prospective fungus was grown on PDA for seven days using the slide culture method [125]. After adding lactophenol cotton blue, the mycelia were found under a microscope. To identify fungi, hyphae and conidia’s morphological characteristics were utilized.

### 3.4. Identification of Fungi Using a Molecular Approach

By Sigma Scientific Services Co., genomic DNA was extracted. To amplify the ribosomal ITS region, ITS 1 (5′-TCC GTA GGT GAA CCT GCG G-3′) and ITS 4 (5′-TCC TCC GCT TAT TGA TAT GC-3′) were utilized as forward and reverse primers, respectively. The following conditions applied to thermal cycling: ten minutes of initial denaturation at 95 °C, thirty seconds of denaturation at 95 °C, one minute of annealing at 57 °C, and one minute of extension at 72 °C were all included. One cycle of post-cycling expansion was performed for ten minutes at 72 °C. Following the manufacturer’s instructions, the PCR yields were purified using the GeneJET PCR Purification Kit (Thermo K0701, Waltham, MA, USA), and the refined DNA was then stored. The PCR yields were purified using the GeneJET PCR Purification Kit (Thermo K0701, Waltham, MA, USA) under the manufacturer’s instructions, and the refined DNA was thereafter stored at −20 °C. Ultimately, an ABI 3730xl DNA sequencer was used to sequence the improved PCR result. The final gene product sequence of the fungal isolate was aligned using NCBI BLAST (Basic Local Alignment Search Tool; http://blast.ncbi.nlm.nih.gov/; accessed on 14 November 2023) against sequences that were already available in the GenBank database. Using the MEGA 5 program, the phylogenetic tree was constructed using the neighbor-joining strategy. The isolate sequence that was found was entered into the GenBank database and given an entry number [126].

### 3.5. Fermentation in Solid-State Media and Extraction of the Fungi Metabolites

In 1L Erlenmeyer flasks sealed with cotton and autoclaved at 121 °C for 20 min, 100 g of rice combined with 120 mL of sterilized water was used to create a solid rice medium for mass manufacturing. Fifteen solid rice flasks were inoculated with plugs from PDA fungal cultures, and the cultures were left to develop for twenty-one days at room temperature.

### 3.6. Preparation of Ethyl Acetate Fungal Extract

Ethyl acetate (EtOAc) (4 × 300 mL) was used to extract fungal metabolites to exhaustion, as per the instructions [127]. Briefly, the fermentation process was terminated by the addition of ethyl acetate, the process was repeated 4 times and the pooled ethyl acetate extracts of the fungal material were evaporated under a vacuum resulting in a brown residue (FEA). In this investigation, the conventional procedure (solid-state fermentation) was utilized for large-scale fermentation and isolation of significant amounts of the chemicals of interest. VanderMolen et al. [128] suggested that solid media usually yield cultures that are one to two times higher in mass than those cultivated on liquid media.

### 3.7. Liquid Chromatography-Mass Spectrometry Analysis (LC/MS)

Using methanol HPLC grade, the ethyl acetate extract was dissolved and filtered by a membrane disc filter 0.2 μm, then into an RP C-18 column 5 µm, 125 mm × 4 mm, 10 μL of the sample was injected. Gradient elution was employed, with a flow rate of 0.2 mL/min. The total program time is 34 min. Mass spectra were detected in the ESI negative ion mode: source temperature 150 °C, cone voltage 30 eV, capillary voltage 3 KV, desolvation temperature 440 °C, cone gas flow 50 L/h, and desolvation gas flow 900 L/h. 

### 3.8. Antimicrobial Screening

The screening of antibacterial and antifungal activities for FEA was conducted using the disk diffusion method, following the standard CLSI procedure [129]. Test microbes included two Gram-positive bacteria (*S. aureus* ATCC 25923; MSSA) and MRSA ATCC-700788), two Gram-negative bacteria (*E. coli* ATCC 25922 and *P. aeruginosa* ATCC9027), and one yeast strain (*C. albicans* ATCC 10231). FEA (20% *w*/*v*) was tested using bacterial and yeast suspensions adjusted to a turbidity equivalent to 0.5 McFarland (1.5 × 10^8^ CFU/mL) in Trypticase Soy Broth (TSB). The prepared disks were placed on Muller–Hinton Agar, with DMSO disks as negative controls and disks containing antibiotics Vancomycin 10 μg, Gentamicin 10 μg, and Nystatin 10 μg were used as positive controls for Gram-positive, Gram-negative bacteria and yeast, respectively. After incubation at 37 °C for 24 h, zones of inhibition were determined according to [130,131].

### 3.9. Determination of Minimum Inhibitory Concentrations

FEA underwent further testing to determine its MIC against sensitive isolates employing the agar well diffusion method [131]. Various concentrations (10, 5, 2.5, 1.25, 0.6, and 0.3%) of the extract were meticulously prepared using a two-fold serial dilution. For each concentration, 1 mL of the prepared inoculum of sensitive isolates (log phase) was pipetted into sterile Petri dishes, followed by the addition of Trypticase Soy agar and thorough mixing. After solidification, wells were created using a sterile cork borer (6 mm in diameter) on agar plates containing the inoculums. Subsequently, 100 μL of the extract dilution was transferred to the respective wells, ensuring that each plate contained only four wells. Following a 30 min refrigeration period, the plates were incubated at 37 °C for 18 h. MIC was defined as the lowest concentration inhibiting the growth of the respective microorganisms. All assays were conducted in triplicate, and DMSO served as a control in these experiments.

### 3.10. Antibiofilm Screening 

To assess the impact of extracts on biofilm formation, sublethal concentrations (75%, 50%, and 25% of MIC) were employed against biofilm-forming sensitive isolates, namely *S. aureus* ATCC 25923 (MSSA) and MRSA ATCC-700788 [132]. 

#### 3.10.1. Inhibition of Biofilm Formation–Prevention of Initial Bacterial Cell Attachment

The potential of FEA to impede initial cell attachment was explored through the biofilm inhibition assay [133]. In brief, 100 μL of a standardized concentration of cultures with OD_560_ = 0.02 (1.0 × 10^6^ CFU/mL) was added to individual flat-bottomed 96-well microtiter plates and incubated at 37 °C for 4 h without shaking. Subsequently, the plates were removed from the incubator, and 100 μL aliquots of the extract were added in triplicate to the wells, resulting in final sub-MIC concentrations (75%, 50%, and 25% of MIC). The plates were then further incubated at 37 °C for 24 h without agitation. DMSO served as the negative control. The biomass was quantified using the modified crystal violet staining method.

#### 3.10.2. Inhibition of Development of Pre-formed Biofilms–Assessment of Destruction of Biofilm Mass

FEA was assessed for its ability to induce the destruction of pre-formed biofilms according to the method performed by Famuyide et al. (2019) [52]. A 100 μL aliquot of a standardized concentration of tested cultures with OD_560_ = 0.02 (1.0 × 10^6^ CFU/mL) was added to individual flat-bottomed 96-well microtiter plates and incubated at 37 °C for 24 h without shaking to allow for the development of a multilayer biofilm. Subsequently, 100 μL aliquots of the extract or its fractions were added to the wells of a 96-well microtiter plate, achieving final sub-MIC concentrations (75%, 50%, and 25% of MIC), and the plates were further incubated at 37 °C for 24 h. The incubation was conducted without agitation. DMSO served as the negative control. The biomass was quantified using the modified crystal violet staining method [133].

#### 3.10.3. The Crystal Violet Staining Assay

The assay followed the method outlined by Famuyide et al. (2019) [52]. Briefly, 96-well microtiter plates were washed five times with sterile distilled water, followed by air-drying and oven-drying at 60 °C for 45 min. Subsequently, wells were stained with 100 μL of 1% crystal violet and incubated at room temperature for 15 min. After three washes with sterile distilled water, a semi-quantitative assessment was conducted by destaining with 125 μL of 30% acetic acid solution for 10 min at room temperature. A 100 μL aliquot of the destaining solution was transferred to a new sterile plate, and absorbance at 590 nm was measured using a microplate reader (BioRad). The percentage inhibition of biofilm was calculated based on the mean absorbance of the samples using the equation below [53].

$$Percentage\;Inhibition=\frac{OD\;negative\;control-OD\;experiment}{OD\;negativ\;econtrol}*100$$

### 3.11. Antiviral Activity 

In this study, Nawah-Scientific, Egypt, provided the Low Pathogenic Corona Virus (229E) and Vero E6 cells. Vero E6 cells were cultured in DMEM medium supplemented with 10% fetal bovine serum and 0.1% antibiotic/antimycotic solution, with reagents sourced from Gibco BRL. Antiviral and cytotoxicity assays were conducted using the crystal violet method [134,135]. Briefly, Vero E6 cells were seeded one day before infection, and the infectivity of the Low Pathogenic Corona Virus (229E) was determined by monitoring cytopathic effects (CPE) and calculating cell viability percentages.

For the antiviral activity assessment, a 96-well culture plate was used, and 0.1 mL of diluted virus suspension was added to cells along with various concentrations of test compounds. The culture plates were then incubated, and the development of CPE was monitored. After staining and quantification, the antiviral activity was calculated using the Pauwels et al. (1988) equation [136], allowing for the determination of the 50% CPE inhibitory dose (IC_50_).

To evaluate cytotoxicity, cells were seeded in a 96-well plate, treated with serially diluted samples, and incubated. After the incubation period, cells were processed similarly to the antiviral assay, and the 50% cytotoxic concentrations (CC_50_) were determined. CC_50_ and IC_50_ were calculated using GraphPad PRISM Version 5.01 software.

### 3.12. Research on ADME (Absorption, Distribution, Metabolism, and Excretion) and Pharmacokinetics

The Absorption, Distribution, Metabolism, and Excretion (ADME) and Pharmacokinetic Studies were carried out using SWISSadme [137] (Swiss Institute of Bioinformatics online source), link: http://www.swissadme.ch accessed on 1 October 2023, to determine whether the compounds had the potential to be a promising pharmaceutical drug. By using the Swiss ADME (http://www.swissadme.ch/; accession date: 18 February 2024) molecules’ bioavailability radar, the physicochemical properties of the identified compounds for oral bioavailability were determined. The pink area represents the optimal ranges for the represented compound’s oral bioavailability based on six physicochemical characteristics: polarity, size, solubility, lipophilicity, flexibility, and saturation. To predict the compound’s blood barrier and GIT absorption, the Boiled Egg approach was also used.

### 3.13. In Silico Studies (Molecular Docking-Coupled Dynamics Simulations)

The probable molecular binding mode between the identified compounds and different enzymes involved in the antimicrobial and antiviral activity was evaluated using the CDOCKER algorithm in Discovery Studio 4.5. (Accelrys Software, Inc., San Diego, CA, USA). The crystal structures of several different protein targets were obtained using the Protein Data Bank (http://www.rcsb.org/pdb/; accession date: 19 February 2024). The enzymes are *S. aureus* teichoic acid-associated β-glycosyltransferase enzyme (TarS; PDB = 5tzj) evaluating the antibiofilm activity; MurE ligase (PDB = 4c12), penicillin-binding proteins (PBP2a; PDB = 3zg0) for assessing the antibacterial activity; and finally HCoV-229E main protease (PDB = 7yrz) which demonstrates the antiviral activity. The protein was refined after the water molecules were eliminated. For each tested enzyme, the binding of the co-crystallized inhibitor and the target enzyme served as the basis for identifying the binding site. Rule-based docking was used to dock all identified compounds and the specific ligand for each enzyme into the protein-binding site, after the co-crystallized ligand was removed. The interaction energy was calculated to examine how the ligand molecules and receptors interacted. The best ligand-binding poses were chosen by sorting the top 10 ligand-binding poses for each ligand according to their CDOCKER interaction energies and looking at the predicted binding interactions.

Best docked complex model for each compound proceeded through molecular dynamics simulations using GROMACS-19 under CHARMM36m and CHARMM-General forcefields following solvation within the TIP3P-water model under periodic boundary conditions [138]. Models were ionized at physiological pH = 7.4 and neutralized using a sufficient number of chloride and potassium ions. System minimization was performed by steepest-descent algorithm-minimization steps (5 ps), then equilibrated at NVT followed by NPT ensembles for 500 ps each [104,138]. Systems were produced for 100 ns molecular dynamics simulations under the NPT ensemble and far-range electrostatic interactions were computed using Particle-Mesh/Ewald algorithm [139]. Root-mean-square deviations (RMSDs_Å) and RMS-fluctuations (RMSFs_Å) were monitored regarding the entire trajectories, while free-binding energies of compound-NCAPG-kleisin complexes were estimated via Molecular Mechanics/Poisson-Boltzmann (MM-PBSA_kJ/mol) single-trajectory approach [115]. Visualizing the simulated complexes at specified timeframes as well as conformational analysis were performed using PyMOL 2.0.6 software.

## 4. Conclusions

This study represents a sustainability approach for fruit peels, which are regarded as industrial waste. Peels can be used as a valuable source of endophytic fungi to enhance their economic value. The isolated *A. flavus* is an endophytic fungus that has significant secondary metabolites and owns a selective antibacterial and antibiofilm potential against Gram-positive microorganisms such as MSSA and MRSA; in addition, it exhibited a promising antiviral activity. The promising computational findings encourage deeper biological in vivo experiments for the identified metabolites which can be used singly, in combination, or in addition to presently prescribed antibiotics to increase their effectiveness and lessen the microbes’ resistance. Additional research is recommended to assess the potential of this promising endeavor across diverse clinical bacterial strains, with a particular emphasis on further exploration concerning coronaviruses, particularly the recently emerged SARS-CoV-2. It is also advised that more research be conducted to identify the various endophytic fungal species that are concealed within fruit peels, as well as their secondary metabolites, modes of action, and biological activities.

## Figures and Tables

**Table 1:** Morphological and microscopical description of *A. flavus*.

Morphological Characters		Microscopic Characters	
**Surface**	Yellowish-black	**Hyphae**	Thread-like septate branched
**Margins**	Entire	**Conidia**	Olive green (4 to 7 μm), roughened
**Reverse side**	Greenish-yellow	**Phialides**	uniseriate and biseriate phialides
**Growth**	Moderate		
**Elevations**	Umbonate		

**Table 3:** Antimicrobial activity—Zones of inhibition for FEA of *Aspergillus flavus* against tested isolates.

	Inhibition Zone Diameter (mm)				
Bacterial Strains		Negative Control	Positive Control		
	FEA	DMSO	Vancomycin	Gentamicin	Nystatin
*S. aureus* ATCC 25923 (MSSA)	15 ± 0.4	0	18 ± 0.2	–	–
MRSA ATCC-700788	11 ± 0.7	0	13 ± 0.3	–	–
*E. coli* ATCC 25922	0	0	–	19 ± 0.7	–
*P. aeruginosa* ATCC9027	0	0	–	25 ± 1.1	–
*C. albicans* ATCC 10231	0	0	–	–	15 ± 0.5

All measurements were conducted in triplicate, and the results are presented as means ± standard deviation (SD).

**Table 4:** Lipinski’s rule of five for ADME analysis of the identified compounds.

No.	Compound	M. wt.	Lipophilicity Log Po/w(MLOGP)	Hydrogen Bond Donors	Hydrogen Bond Acceptors	No. of Rule Violations	Drug Likeness
		Less than 500 g/mol	Less than 5	Less than 5	Less than 10	Less than 2 Violations	Lipinski’s Rule Follows Rule
1	Heptelidic acid	280	1.60	1	5	0	Yes
2	Ferulic acid	194	1.00	2	4	0	Yes
3	Oleic acid	282	4.57	1	2	0	Yes
4	Paxilline	435	2.58	3	4	0	Yes
5	Indole	117	1.57	1	0	0	Yes
6	Orientin	448	−2.51	8	11	2	No
7	Kojic acid	142	−1.69	2	4	0	Yes

**Table 5:** CDOCKER interaction energies for the identified phytochemicals at the binding sites of both bacterial and viral biotargets.

Compounds	Designated Targets			
	*S. aureus* MurE (PDB; 4c12)	*S. aureus* PBP2a (PDB; 3zg0)	*S. aureus* TarS (PDB; 5tzj)	*HCoV-229E* Mpro (PDB; 7yrz)
Orientin	−51.75	−49.69	−37.48	−50.15
Heptelidic acid	−49.58	−42.93	−34.54	−39.71
Paxilline	−43.45	−40.51	−31.21	−26.44
Ferulic acid	−39.22	−38.56	−20.76	−25.21
Kojic acid	−22.18	−14.11	−13.23	−19.49
Oleic acid	−21.24	−36.54	−20.15	−21.23
Reference	−54.87	−51.58	−33.67	−69.75

## Data Availability

Data are available within the article.

## Supplementary Materials

The following supporting information can be downloaded at: https://www.mdpi.com/article/10.3390/ph17050656/s1, Figure S1: Negative ionization mode chromatogram representing the major compounds identified from the fungal ethyl acetate extract numbered according to their relative abundance where (1) heptelidic acid, (2) ferulic acid, (3) oleic acid, (4) paxilline, (5) indole, (6) orientin, and (7) kojic acid.

## Abbreviations

HCoV 229E: human coronavirus 229E; ITS-rDNA: internal transcribed space; UPLC/MS: ultra-high performance liquid chromatography-MS; CPE: cytopathic effects; MRSA: methicillin-resistant *Staphylococcus aureus*; MSSA: methicillin-susceptible *Staphylococcus aureus*; ADME: absorption, distribution, metabolism, and excretion; UV: ultra violet; IC_50_: half-maximal inhibitory concentration; ARIs: acute respiratory infections; ATCC: American Type Culture Collection; CC_50_/IC_50_: cytotoxicity (CC_50_) and inhibition concentration (IC_50_); BBB: blood–brain barrier; POLAR, LIPO, INSOLU, and IN-SATU: polarity, lipophilicity, solubility, and saturation; TPSA: topological polar surface area; WLOGP: the atomic log *p*; HCoV-OC43: human coronavirus OC43; UDP-GluNAc: uridine diphosphate *N*-acetylglucosamine; RMSD: Root Mean Square Deviation; PBP2a: penicillin-binding protein.
