# Supplementary material for: Unveiling the Multifaceted Capabilities of Endophytic Aspergillus flavus Isolated from Annona squamosa Fruit Peels against Staphylococcus Isolates and HCoV 229E—In Vitro and In Silico Investigations
Source: Pharmaceuticals (Basel). 2024 May 19;17(5):656. doi: 10.3390/ph17050656 (PMC11124496; doi:10.3390/ph17050656)
Supplement: Supplementary file 1 [file pharmaceuticals-17-00656-s001.zip › pharmaceuticals-2980897-supplementary.pdf]

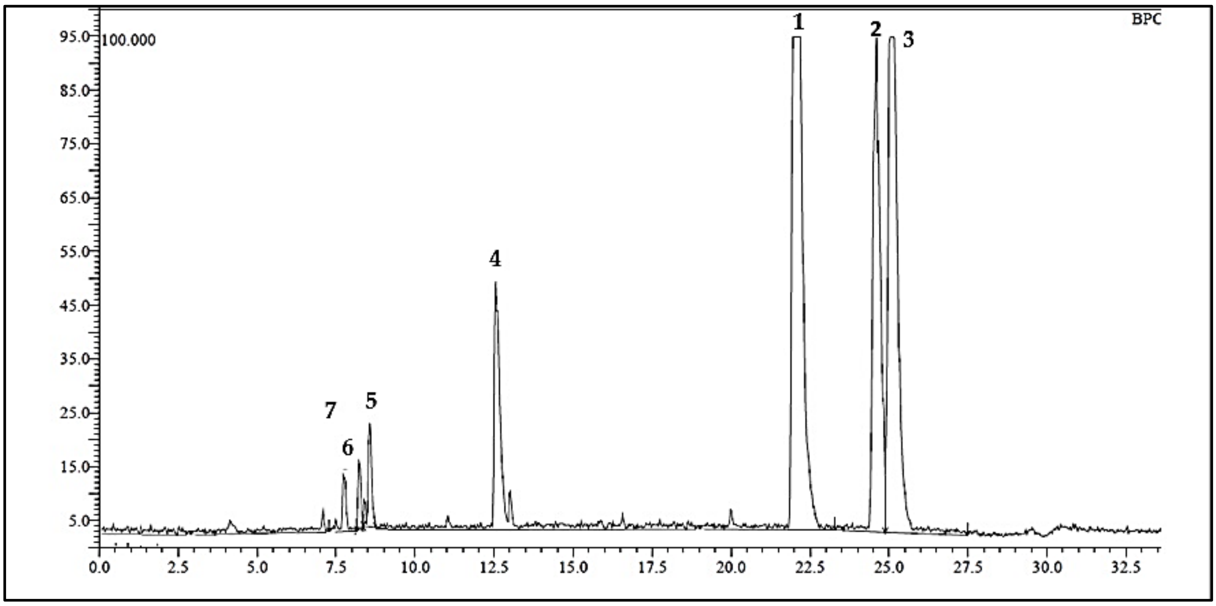


Figure S1: Negative ionization mode chromatogram representing the major compounds identified from the fungal ethyl acetate extract numbered according to their relative abundance where 1) heptelidic acid, 2) ferulic acid, 3) oleic acid, 4) paxilline, 5) indole, 6) orientin and 7) kojic acid.
